# Interplay between bacterial 5′-NAD-RNA decapping hydrolase NudC and DEAD-box RNA helicase CsdA in stress responses

**DOI:** 10.1128/msystems.00718-23

**Published:** 2023-09-14

**Authors:** Milda Mickutė, Renatas Krasauskas, Kotryna Kvederavičiūtė, Gytė Tupikaitė, Aleksandr Osipenko, Algirdas Kaupinis, Monika Jazdauskaitė, Raminta Mineikaitė, Mindaugas Valius, Viktoras Masevičius, Giedrius Vilkaitis

**Affiliations:** 1 Institute of Biotechnology, Life Sciences Center, Vilnius University, Vilnius, Lithuania; 2 Institute of Biochemistry, Life Sciences Center, Vilnius University, Vilnius, Lithuania; 3 Thermo Fisher Scientific Baltics, Vilnius, Lithuania; 4 Institute of Chemistry, Faculty of Chemistry and Geosciences, Vilnius University, Vilnius, Lithuania; University of California San Diego, La Jolla, California, USA

**Keywords:** RNA modification, RNA hydrolase, DEAD-box helicase, RNA cap, NAD-RNA

## Abstract

Both prokaryotic and eukaryotic RNAs can be 5′-capped by the metabolite nicotinamide adenine dinucleotide (NAD). Nudix hydrolases, such as bacterial NudC, specifically remove NAD-caps; however, the molecular and cellular functions of these epitranscriptomic modulators remain elusive. Here, we discuss the roles of NudC under stress conditions and the effects of extracellular cues on the NAD epitranscriptome. Our proteome-wide analysis detected the proteins associated with the RNA degradosome as well as ribosomes and stress-responsive proteins in a NudC interactome. Moreover, we confirmed the physical association of NudC with the cold shock DEAD-box RNA helicase CsdA and the RNA chaperone Hfq. Interestingly, knocking out *csdA* similar to ∆*nudC* leads to an increased number of identified 5′-NAD-RNA species compared to wild type, exposing CsdA as a new player in a potentially unexplored layer of NAD-epitranscriptomic landscape. Mass spectrometry analysis also revealed the drastic up-regulation of 5′-NAD-RNA in response to cold. Furthermore, the inactivation of NudC in bacteria changes the levels of sRNA and protein-coding transcripts associated with bacterial chemotaxis and flagellar assembly pathways. We experimentally demonstrate that the decapping hydrolase NudC suppresses the flagellar movement, while CsdA stimulates it. Thus, the interplay between NudC and CsdA regulates bacterial mobility and coordinates stress-avoidance behavior.

**IMPORTANCE:**

Non-canonical 5′-caps removing RNA hydrolase NudC, along with stress-responsive RNA helicase CsdA, is crucial for 5′-NAD-RNA decapping and bacterial movement.

## INTRODUCTION

A nicotinamide adenine dinucleotide (NAD^+^) cap represents a common 5′-terminal RNA modification that is conserved across different species, ranging from Gram-negative and Gram-positive bacteria to yeast, plants, and humans (1). In contrast to the well-known protective 7-methylguanosine (m^7^G) cap that protects mature eukaryotic mRNAs from degradation, NAD-cap can promote both RNA stabilization in bacteria (2, 3) and RNA decay by DXO/Rai1 family enzymes in yeasts and plants/human cells, respectively (4 – 6). Over the last few years, it has been shown that 5′ termini can also be coupled with another metabolic nucleoside polyphosphate, flavin adenine dinucleotide (FAD), 3′-desphospho-coenzyme A (dpCoA), dinucleoside polyphosphates (Np_n_N) as well as uridine diphosphate (UDP) sugars (7 – 10), suggesting a possible layer of metabolite-mediated epitranscriptomic regulation of gene expression and *vice versa*. Indeed, it was demonstrated that disulfide stress induces protective nucleoside tetraphosphate capping of RNAs in *Escherichia coli*, whereas decapping triggers rapid RNA degradation (7). Since bacteria lack a canonical eukaryotic m^7^G-type cap, the ubiquitous redox coenzyme NAD (43% of all caps except Np_n_N) and the precursor of muramic acid in bacterial cell walls, UDP-N-acetylglucosamine (49% of all caps except Np_n_N), appear to be the predominant 5′-modifications for adenosine- or uridine-initiated nascent transcripts in *E. coli*, respectively (10), as well as stress-related Np_n_N (e.g., m7Gp_4_Gm) alarmones detected in a comparable amount (9). Growth phase-dependent alterations in total RNA NAD^+^-capping as well as significant variations in the modification of individual transcripts under the different growth phases detected in *E. coli* bacteria indicated that the protective modification of certain RNA pools could be important to swiftly adapt cells to physiological changes (11). However, the biological roles of NAD^+^-cap on RNA and how internal intake or stress factors impact the (de)NADding processes have remained unclear.

A few families of structurally dissimilar 5′-NAD-RNA decapping (deNADding) enzymes have been identified, including nucleoside diphosphate linked to another moiety X (Nudix) hydrolases generating the nicotinamide mononucleotide and 5′-monophosphorylated RNA in bacteria or mammals (class II deNADding enzymes) (2, 3, 12) and DXO/Rai1 proteins catalyzing the removal of the intact NAD^+^ moiety in yeasts, plants, and humans (class I deNADding enzymes) (4, 5). Deletion of their coding genes causes a substantial boost in global NAD-capped RNA levels, demonstrating that decapping enzymes actively modulate the genome-wide composition of cellular 5′-NAD-RNA (4 – 6, 13). Notably, recent studies suggest that each of two mammalian deNADding enzymes, Nudix hydrolase Nudt12 and DXO, modulates individual subsets of NAD^+^-capped RNA, changing RNA modification levels under glucose deprivation and heat-shock stress conditions, respectively (12). In *Arabidopsis*, the phytohormone abscisic acid-mediated remodeling of NAD^+^ transcriptome is independent of DXO1, indicating that there are additional factors regulating the turnover of specific subsets of 5′-NAD-RNA in response to environmental signals and stresses. Interestingly, even small amounts of 5′-NAD-RNA are sufficient to activate RDR6-dependent post-transcriptional gene silencing (5).

In *E. coli*, the NAD^+^ moiety appears to protect the 5′ termini of RNA, thus enhancing cellular RNA stability (2). Nevertheless, the significance of NAD^+^-cap as well as the effect of deNADing enzymes on bacterial biological function and physiology have remained elusive. To address this lack of knowledge, we examined changes in the 5′-NAD-RNA level under cold shock. Then, we analyzed the interactome of bacterial NudC hydrolase, the enzyme that removes protective NAD^+^, NADH, and dpCoA 5′-caps (8, 14), at ambient and low temperatures. Because bacteria rely on RNA-binding proteins and their complexes to post-transcriptionally adjust gene expression, the identification of putative NudC interaction partners and ultimately multiprotein complexes can have general implications for the understanding of deNADding regulation and/or mechanisms needed for the adaptation of bacteria to altered environmental conditions. Among others, we found an association of NudC with the cold shock DEAD-box RNA helicase CsdA (15) and the RNA chaperone Hfq (16), suggesting that the deNADing protein may play a role in modulating RNA landscapes and/or protein synthesis and thus potentially improve cell fitness in stress conditions. We used NAD captureSeq (∆*nudC* and ∆*csdA* strains) and RNA sequencing (∆*nudC* strain) to illuminate the presence of hitherto unknown NudC- and CsdA-mediated mechanisms regulating cellular epitranscriptomic and transcriptomic profiles. Finally, we showed that NAD capping and the abundance of individual sRNAs could be dynamically regulated in response to environmental and genetical changes, thus affecting these processes, and discovered the link between bacterial motility and the RNA-demodifying NudC hydrolase.

## RESULTS

### The level of total NAD^+^-capped RNA elevates under cold shock conditions in *E. coli*

**FIG 1:**
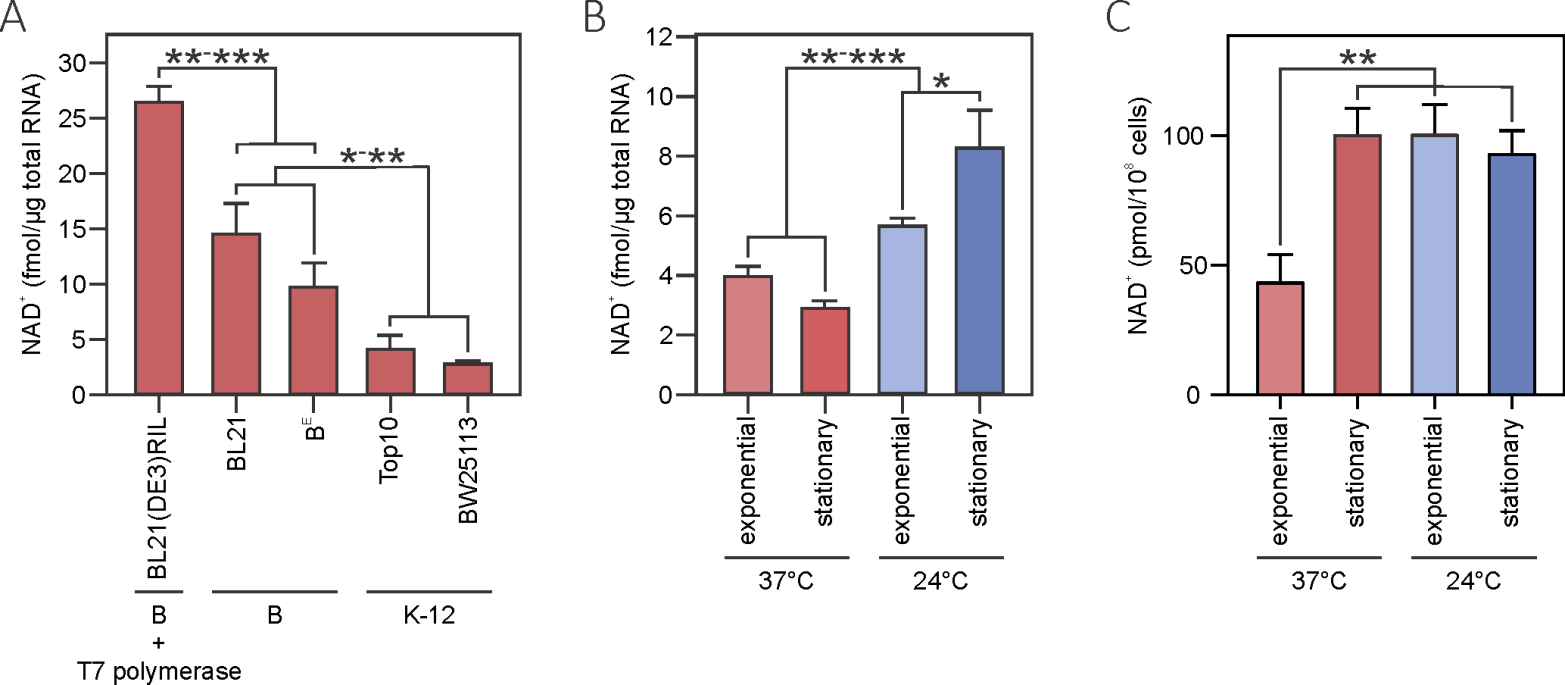
Assessment of the total level of 5′-NAD^+^-RNA modification at ambient and low temperatures in *E. coli*. (**A**) The amount of 5′-NAD^+^-RNA detected in selected *E. coli* B and K-12 strains at the stationary growth phase at 37°C using HPLC-MS/MS analysis. The levels of 5′-NAD^+^-RNA (**B**) and cellular NAD^+^ (**C**) in the BW25113 strain at different growth phases and temperatures. All experiments were performed in triplicate. The significance was calculated using the Welch *t*-test and pairwise comparisons, *P < 0.05, **P < 0.01, ***P < 0.001.

Previous studies using individual *Escherichia coli* K-12 strains, DH5α, Top10, or MG1655, have revealed considerably divergent amounts of 5′-NAD-RNA, ranging from 2.2 to 120 fmol/μg of total RNA (10, 13, 17). Still, it is difficult to directly compare the RNA modification levels reported by different groups, which use diverse experimental conditions and detection methods. To assess whether the amount of total NAD^+^-capped RNA differs among various *E. coli* strains belonging to two distinct phylogenetic subgroups at the stationary phase of growth (Fig. S1), we estimated the amount of NAD^+^ conjugated to RNA by high-performance tandem liquid chromatography-mass spectrometry (HPLC-MS/MS) after total cellular RNA treatment with nuclease P1 in two K-12 derivatives, BW25113 and TOP10, and three widely used B lineages, B^E^, BL21, and BL21(DE3)RIL (Fig. 1A; Fig. S2; Table S1). We found that the extent of NAD^+^-capped RNA is 2–5-fold higher in B strains except BL21(DE3)RIL (9.7–14.5 fmol/µg of total RNA) than in K12 (2.9–4.2 fmol/µg of total RNA), suggesting that common ancestry rather than further mutations impacts the modification level. Notwithstanding the close genetic relationship (18), a large difference was detected between BL21 and its descendant BL21(DE3)RIL, which contains the λDE3 prophage and carries an IPTG-inducible gene for T7 RNA polymerase. The leakage expression of the bacteriophage polymerase (19) may likely lead to the elevated synthesis of 5′-capped RNA in this strain. Indeed, a previous *in vitro* study had demonstrated that single polypeptide T7 polymerase initiated RNA synthesis with NAD^+^ substantially more efficiently than multi-subunit *E. coli* polymerase (19).

A detailed analysis of modification changes during stress conditions was performed using BW25113, which is the parent strain for the *E. coli* K-12 Keio collection of single gene knockouts (20). We quantified the total amount of NAD^+^ moieties on RNA during the active bacterial growth, exponential phase, and under starvation conditions, the late stationary phase, at optimal and low temperatures (Fig. 1B; Fig. S1; Table S1). We observed a marked elevation in the yield of NAD^+^-capped RNA when the temperature was lowered from 37°C to 24°C. Stationary-phase bacteria exposed to cold contained nearly 3-fold higher amount of modified RNA than cultures incubated at 37°C, suggesting that 5′-NAD-RNA could be regulated by extracellular cues. Notably, when the stationary phase of growth was compared to the exponential, a substantial increase in NAD^+^ capping was observed only in BW25113 cells grown at a lower temperature. In contrast to a previous study that reported phase-dependent changes in MG1655 (increased modification levels at the stationary phase) using genome-wide RNA sequencing (11), our HPLC-MS/MS analysis displayed negligible variation at 37°C. Thus, it is plausible that NAD^+^-capping/decapping could be differently controlled in certain *E. coli* strains.

It was shown that NAD/NADH caps are incorporated during the transcription initiation of bacterial and eukaryotic RNA when an RNA polymerase uses the metabolic nucleotide in place of ATP (8). The efficiency of this modification depends on the [NADH]/[ATP] ratio *in vitro* (8), and the 5′-NAD-RNA level appears to correlate with the intracellular concentration of free NAD(H) in human HEK293T cells *in vivo* (13, 19). The free NAD^+^ concentration measured by the NAD^+^/NADH quantification kit remained constant throughout the growth cycle of *E. coli* BW25113 cells at low temperatures and was similar to that of the stationary phase at 37°C. The level of NAD-capped RNA increased up to ~3-fold in cold-exposed bacteria (Fig. 1B and C; Table S1), suggesting that intracellular NAD^+^ homeostasis is not a conclusive factor in regulating the level of modified RNA during cold stress in these bacteria. Supporting this assumption, the level of NAD^+^-capping was not affected despite a 2-fold reduction in free NAD^+^ amount at the exponential phase compared to the stationary phase at 37°C. Alltogether, these findings imply post-transcriptional control of 5′-NAD-RNA abundance in response to stresses.

### DEAD-box RNA helicase CsdA is an interaction partner of the 5′-NAD-RNA decapping protein NudC

**FIG 2:**
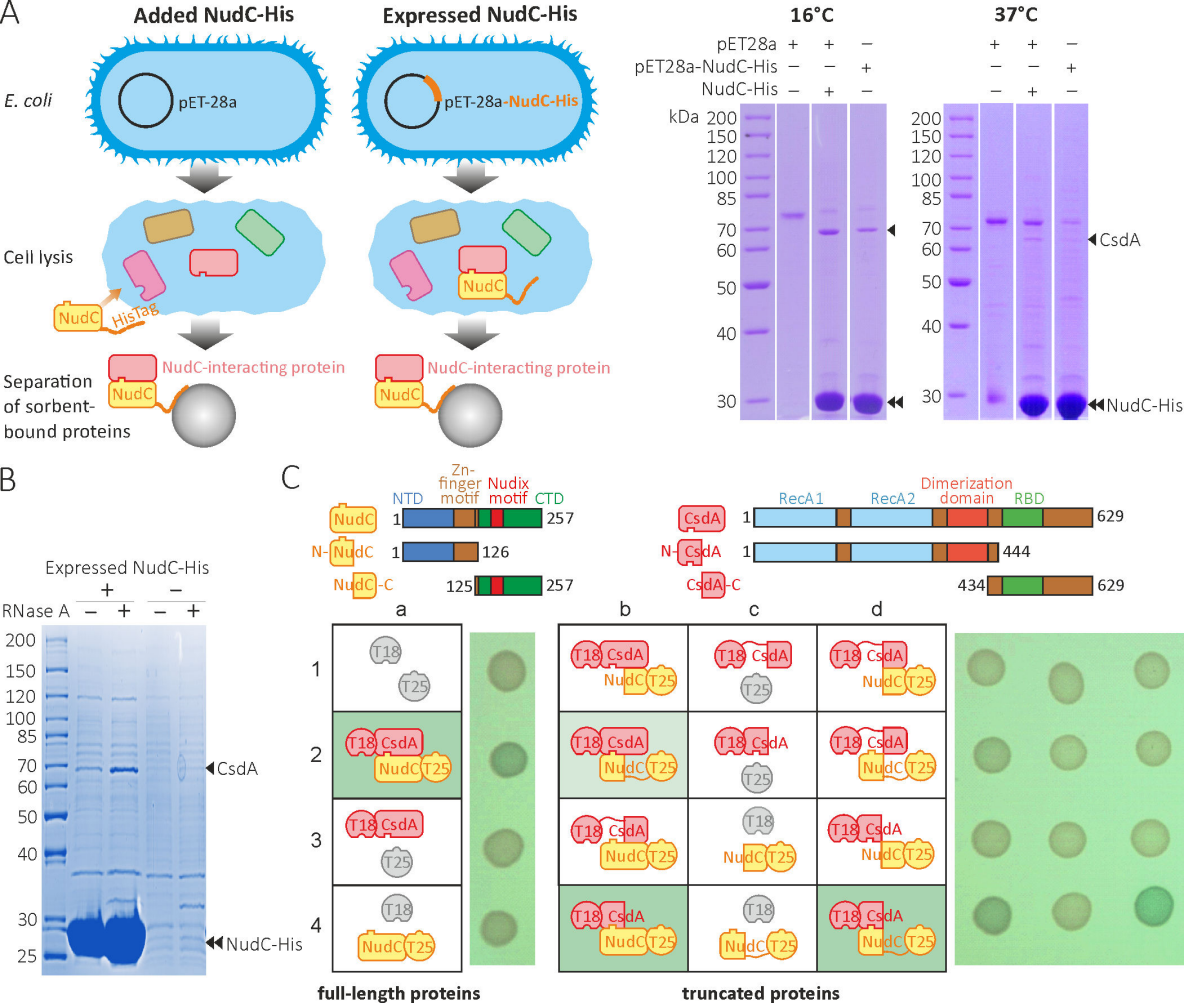
Bacterial NudC hydrolase interacts with the DEAD-box helicase CsdA *in vivo* and *in vitro*. (**A**) On the left, an experimental scheme for the isolation of NudC-interacting proteins. Purified NudC-His was either directly added to bacterial lysates or expressed inside the cells. After incubation with Ni^+2^-sepharose sorbent, the beads were washed four times, and proteins that co-purified with NudC-His were separated by SDS-PAGE and analyzed by MS. On the right, the pull-down assays from *E. coli* BL21(DE3)RIL cells grown at 16°C or 37°C. The experiments were repeated independently at least three times, and the representative results are provided. (**B**) The expressed NudC-His from *E. coli* BL21(DE3)RIL directly interacts with CsdA *in vivo* with no need for an RNA intermediate (see Fig. S4B for the experimental scheme). (**C**) The BATCH study of the NudC-CsdA interactions *in vivo* using proteins fused with T18 or T25 adenylate cyclase subunits. Physical interactions between full-length proteins and/or N-terminal regions yielded blue colonies on LB agar plates with X-Gal (**a2, b2, b4, and d4**). Domain structure of CsdA and NudC proteins and their truncated variants are shown schematically at the top: NTD, N-terminal domain; CTD, C-terminal domain; RecA1, RecA2, helicase-specific domains; RBD, RNA-binding domain. At least three independent transformations were made, and representative results are shown.

Nudix hydrolase NudC can efficiently remove the NAD^+^, NADH, and dpCoA caps but does not affect the conventional initiating nucleotides (2, 8). Thus, this enzyme may play a key role in the regulation of 5′-NAD-RNA level during the adaptation of *E. coli* to low temperatures and stress. Because the majority of cellular processes are executed by multicomponent protein assemblies, we first looked for binding partners of polyhistidine-tagged NudC (NudC-His) at 16°C using the affinity-tag pull-down technique (Fig. 2A, left gel). The fusion protein was expressed in the BL21(DE3)RIL strain containing the highest amount of 5′-NAD-RNA (see above), or the recombinant enzymatically active NudC-His (Fig. S3) was added to the soluble fraction of cell lysate. In both samples, we detected a clear band of enriched protein with a molecular mass near 70 kDa on SDS-PAG, which was absent in the control experiment containing a parental vector without an insert. A similar band, though of lower intensity, was also observed in the 37°C assay (Fig. 2A, right gel) and in experiments with the BW25113 strain at 16°C (Fig. S4A). As these bands were discretely cut out and analyzed by mass spectrometry, we identified the DEAD-box RNA helicase CsdA (also known as DeaD) as a potential interactor of NudC. In agreement with our results, the expression of CsdA, which is implicated in various cellular processes at low temperatures, is highly enhanced during cold shock (21). Remarkably, CsdA has been reported to associate with integral components of the RNA degradosome, such as RNase E, establishing the ‘cold shock degradosome’ (22). To assess whether the newly identified interaction is mediated by the common target of both proteins—the RNA molecule, we performed a pull-down experiment with BL21(DE3)RIL cells expressing NudC-His after treatment with RNAse A (Fig. S3B). The presence of the CsdA band on the gel (Fig. 2B) indicates that the formation of a stable NudC-CsdA complex can occur independently of RNA binding. Because the expression of active NudC could potentially induce changes in the levels of NAD-RNAs or free NAD, which might affect protein localization and interaction with binding partners, we constructed the plasmid carrying the NudC catalytic mutant. In this mutant, the glutamic acid (E178) within the Nudix motif was replaced by a glutamine, rendering the hydrolase inactive (23). A pull-down experiment with NudC(E178Q) reconfirmed the NudC-CsdA interaction at low temperature (Fig. S4C), demonstrating that the interaction between NudC and DeaD does not require the hydrolase activity.

To confirm the direct interactions between NudC and CsdA *in vivo*, we applied the bacterial adenylate cyclase-based two-hybrid system (BACTH). We first compared the binding preferences of proteins with T18 or T25 adenylate cyclase subunits fused to different termini of NudC and CsdA (Fig. S5A). Since the T18-NudC/CsdA-T25 pair displayed the strongest positive signal, we used this tagging variant (N-terminal T18 fused to NudC and C-terminal T25 fused to CsdA) to precisely map the NudC and CsdA interaction regions (Fig. 2C; Fig. S6A). Only the N-terminal regions of both proteins revealed robust interaction signals, while both C-terminal constructs failed to bind their partner’s full-length or truncated forms, indicating physical contact between the N-terminal surfaces of NudC and CsdA. The positive interactions were specific since strains containing all protein constructs with their corresponding partner’s empty vector control formed white colonies. The BACH experiment with the truncated N-NudC protein, which lacks the C-terminal domain with the Nudix motif, reaffirms that the formation of the NudC-DeaD interaction does not depend on the catalytic activity of the hydrolase.

### Proteome-wide profiling of the NudC interactome

**FIG 3:**
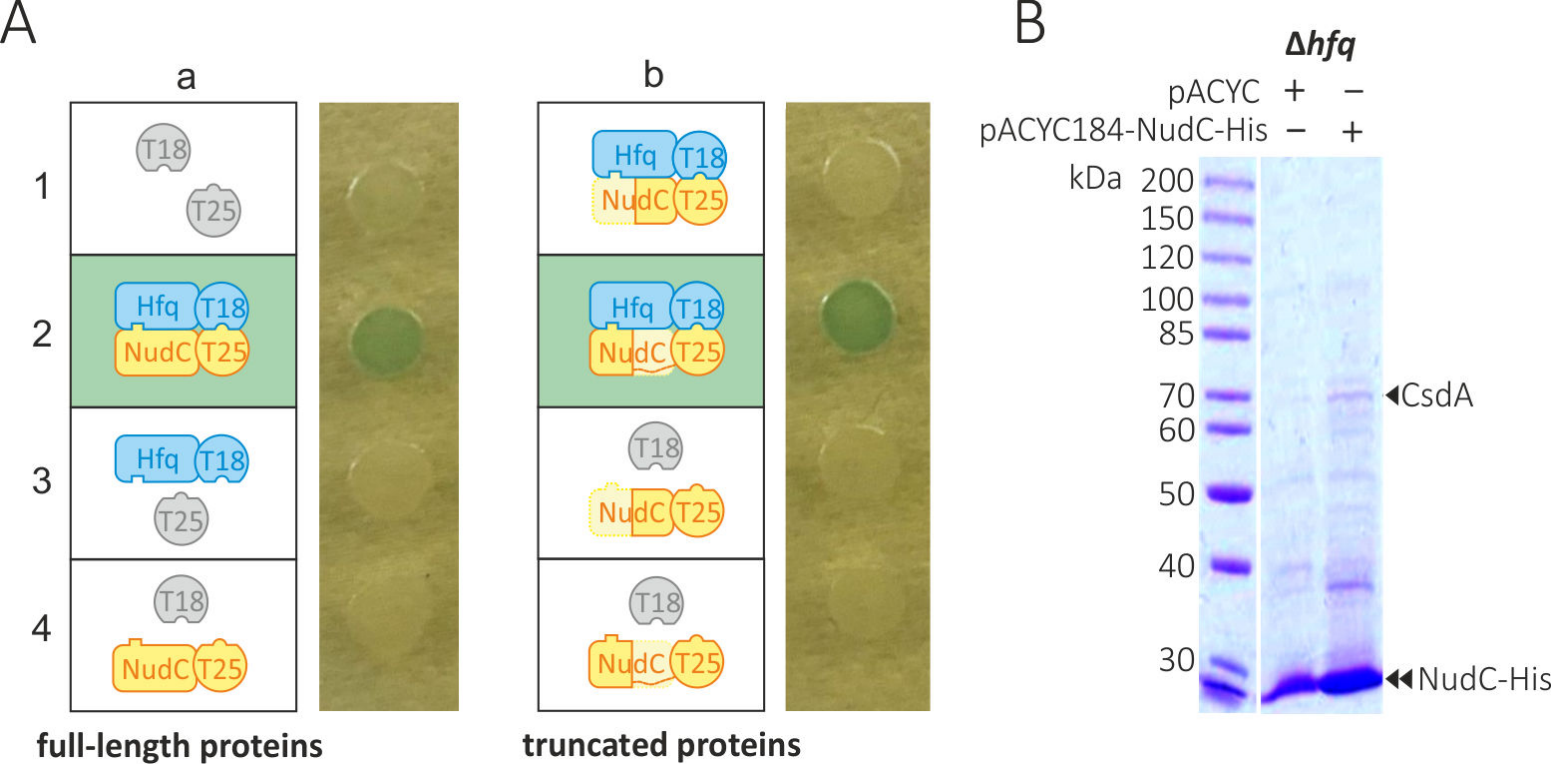
Identification of direct contacts between NudC and the RNA chaperone Hfq. (**A**) NudC forms a complex with Hfq (a2) using its N-terminal domain (b2). At least three independent transformations were made, and representative results are shown. (**B**) Hfq is not essential for the formation of a CsdA-NudC complex *in vivo* since NudC interacts with CsdA in Hfq-deficient JW4130 cells at 16°C.

Apart from CsdA, a couple of additional, though fainter, bands were specifically observed in NudC-His-containing samples (Fig. 2A and B; Fig. S4A), therefore we decided to perform a proteome-wide screen of the NudC interactome using an improved affinity-tag pull-down technique. After washing off proteins not bound to nickel-charged resin, we added 8 M urea and incubated for 10 min at 8°C to elute NudC-associated proteins. Thus, the overwhelming majority of NudC-His and other directly immobilized proteins remained captured on the beads and were omitted from LC-MS^E^-based identification analysis. The interactomes were deciphered by either expressing NudC-His in live cells or adding the purified protein to the *E. coli* BL21(DE3)RIL lysate grown at optimal or cold temperatures. We identified 56 potential NudC interaction partners that could be divided into five major groups (Table 1 and Additional file 1). Reassuringly, we detected CsdA protein in all tested conditions. In addition, a few other proteins co-purified with NudC appeared to be associated with the RNA degradosome: the 3′-exoribonuclease polynucleotide phosphorylase (PNPase), a cold-induced protein essential for *E. coli* and *Bacillus subtilis* growth at low temperatures (22, 24), and the RNA chaperone Hfq, a major post-transcriptional regulator modulating mRNA stability and translation through mechanisms usually mediated by small regulatory RNAs (25). Notably, the resident RNA helicase of the RNA degradosome, RhlB, which is pivotal for promoting the RNA degradation at 37°C, as well as ribonuclease E (RNase E) were not detected, indicating that NudC, together with the aforementioned interaction partners, may perform functions unrelated to the degradosome. The interaction between NudC and Hfq was confirmed by a bacterial two-hybrid analysis (Fig. 3A; Fig S5B and S6). Further BACTH complementation assays using the truncated proteins revealed that the RNA chaperone binds to the N-terminal region of NudC, which has also been shown to bind another RNA degradosome protein, CsdA, in the aforementioned experiments. Nevertheless, NudC-His expressed in an Hfq-deficient strain at low temperature successfully pulled down CsdA (Fig. 3B), suggesting that Hfq does not contribute to the physical interaction between NudC and CsdA proteins *in vivo*.

**TABLE 1:** NudC interactome determined by LC-MS^E^-based identification analysis of proteins captured with externally added or internally expressed His-tagged NudC^*a*^

Functional group	UniProt accession number	Name of interacting protein	Purified NudC-His added to lysate		NudC-His expressed in *E. coli* cells	
			16°C	37°C	16°C	37°C
RNA degradosome	A0A140N4Y7	CsdA helicase	+++	++	+++	++
	A0A140NGK1	RNA chaperone protein Hfq			+	+
	A0A140N5F4	Polyribonucleotide nucleotidyltransferase	++		++	
Stress-response chaperones	A0A140N9U7	Cold-shock chaperone protein YdfK	++		+++	
	A0A140NCD4	UspA domain protein UspG (UP12)		+	++	++
Protein-folding chaperones	A0A140NET2	Trigger factor			+++	+++
	A0A140NFZ9	Chaperone protein DnaJ	+++	+++		++
	A0A140N6A5	Heat-shock chaperone protein ClpB			+	+
	A0A140ND61	Heat-shock chaperone protein HtpG			++	++
	A0A140N1Q5	Small heat shock protein IbpA	++	++		
Transcription and translation	A0A140NF01	Transcription termination factor Rho			+	+
	A0A140NFM7	50S ribosome subunit protein L1	+++	+++	+++	+++
	A0A140N7J1	50S ribosome subunit protein L2	+++	+++	+++	+++
	A0A140N3G7	50S ribosome subunit protein L3^*b*^	+++	+++	+++	+++
	A0A140N5K8	50S ribosome subunit protein L4	+++	+++	+++	+++
	A0A140NDV1	50S ribosome subunit protein L9	+++	+++	+++	+++
	A0A140NDB6	50S ribosome subunit protein L10	++	++		
	A0A140NF32	50S ribosome subunit protein L11	++	++		+
	A0A140N598	50S ribosome subunit protein L13	++	++	++	++
	A0A140N711	50S ribosome subunit protein L15	+++	+++	+++	+++
	A0A140N6Z2	50S ribosome subunit protein L16	++	++	++	++
	A0A140N4M0	50S ribosome subunit protein L17	++	+++	+++	+++
	A0A140N4L0	50S ribosome subunit protein L18	++	++	++	++
	A0A140N6T7	50S ribosome subunit protein L19	+++	+++	+++	+++
	A0A140NBS1	50S ribosome subunit protein L20	++	++	++	++
	A0A140N5D7	50S ribosome subunit protein L21	+++	+++	+++	+++
	A0A140N2S3	50S ribosome subunit protein L22	++	++	++	++
	C6EGE8	50S ribosome subunit protein L23				+
	A0A140N340	50S ribosome subunit protein L27			++	++
	A0A140N3L9	50S ribosome subunit protein L28	++		++	++
	A0A140N7F5	50S ribosome subunit protein L29	++		++	
	A0A140NAZ2	50S ribosome subunit protein L32	++		+++	+++
	A0A140NHV0	50S ribosome subunit protein L34	++	++	++	++
	A0A140NBA5	30S ribosome subunit protein S1	+	+	++	++
	A0A140NFK2	30S ribosome subunit protein S2			+	++
	A0A140N4K1	30S ribosome subunit protein S3	++	++	++	++
	A0A140N548	30S ribosome subunit protein S4	+++	+++	+++	+++
	A0A140N6Z9	30S ribosome subunit protein S5	++	++	++	+++
	A0A140NGG7	30S ribosome subunit protein S6	+	+	++	++
	A0A140N6W8	30S ribosome subunit protein S7			++	++
	A0A140N2Z9	30S ribosome subunit protein S9	+	+	++	++
	A0A140N7L9	30S ribosome subunit protein S11	++	++	++	++
	A0A140N4H3	30S ribosome subunit protein S12^*c*^	++	++	++	++
	A0A140N5B4	30S ribosome subunit protein S13	++	++	++	++
	A0A140N811	30S ribosome subunit protein S15			+	+
	A0A140NGH1	30S ribosome subunit protein S18	+	+	++	++
	A0A140N528	30S ribosome subunit protein S19			++	++
	A0A140NFU3	30S ribosome subunit protein S20	++	++	+++	++
	A0A140N8B4	30S ribosome subunit protein S21	++	++	++	++
	A0A140N3T4	Translation initiation factor IF-2			+	++
Metabolism and other	A0A140N3S1	CRM domain-containing protein			++	++
	A0A140NEF7	Phosphopentomutase		++	++	+
	A0A140N4R6	Uncharacterized protein			+	+
	A0A140N9H5	tRNA/rRNA methyltransferase (SpoU)				+
	A0A140NA80	Succinate dehydrogenase flavoprotein	++	++	+	++
	A0A140NEQ0	Clp protease ATP-binding subunit ClpX			+	+
	A0A140NF86	Protease ATPase subunit HslU			++	++

^*a*^ Only proteins detected in at least two of three biological replicates and not present in control samples without NudC are shown. +, ++, and +++ indicate 0.5–1, 1–5, and 5–100 fmol of protein detected, respectively.

^*b*^ Samples with NudC had nine times higher protein concentrations than the controls.

^*c*^ Samples had four times higher protein concentrations than the controls.

The potential interactome also included two additional stress-responsive proteins—the cold shock chaperone protein YdfK, which is drastically up-regulated in low temperature-shifted bacteria (26), and the universal stress protein UspG (also known as UP12) (27). We hypothesized that the protein-folding chaperones belonging to the third group bind a fraction of misfolded recombinant NudC, although further studies are needed to prove this convincingly.

Roughly two-thirds of the identified proteins (39 out of 57) were ribosome proteins and translation initiation factors, suggesting a possible implication of NudC in translation-related processes (Table 1). The hydrolase extracted about 80% of ribosome proteins that are located in both 50S and 30S subunits, hinting that the NudC-ribosome interaction occurs in a complex that possibly includes a supplementary factor, e.g., RNA or protein, such as CsdA, linked to ribosome biogenesis (28) (Fig. S7).

Taken together, the interactome results show that NudC appears to be implicated in various macromolecular metabolic processes, especially the ones associated with cellular responses to cold.

### NudC localization and phenotypic analysis of cells with impaired *nudC* and *csdA* expression

In an attempt to determine the impact of the NudC and CsdA proteins on cold tolerance, we compared the growth rates of wild-type (wt) and the Nudix hydrolase or the cold shock DEAD-box RNA helicase-lacking bacteria under different temperature conditions. In accordance with a previous report (28), the growth of ∆*csdA* and ∆*csdA*∆*nudC* was inhibited at low temperatures, while ∆*nudC* maintained a normal growth rate (Fig. S1 and S8A). Overexpression of CsdA, but not NudC protein, rescued the growth defect in *∆csdA* and *∆csdA∆nudC* strains (Fig. S8B).

**FIG 4:**
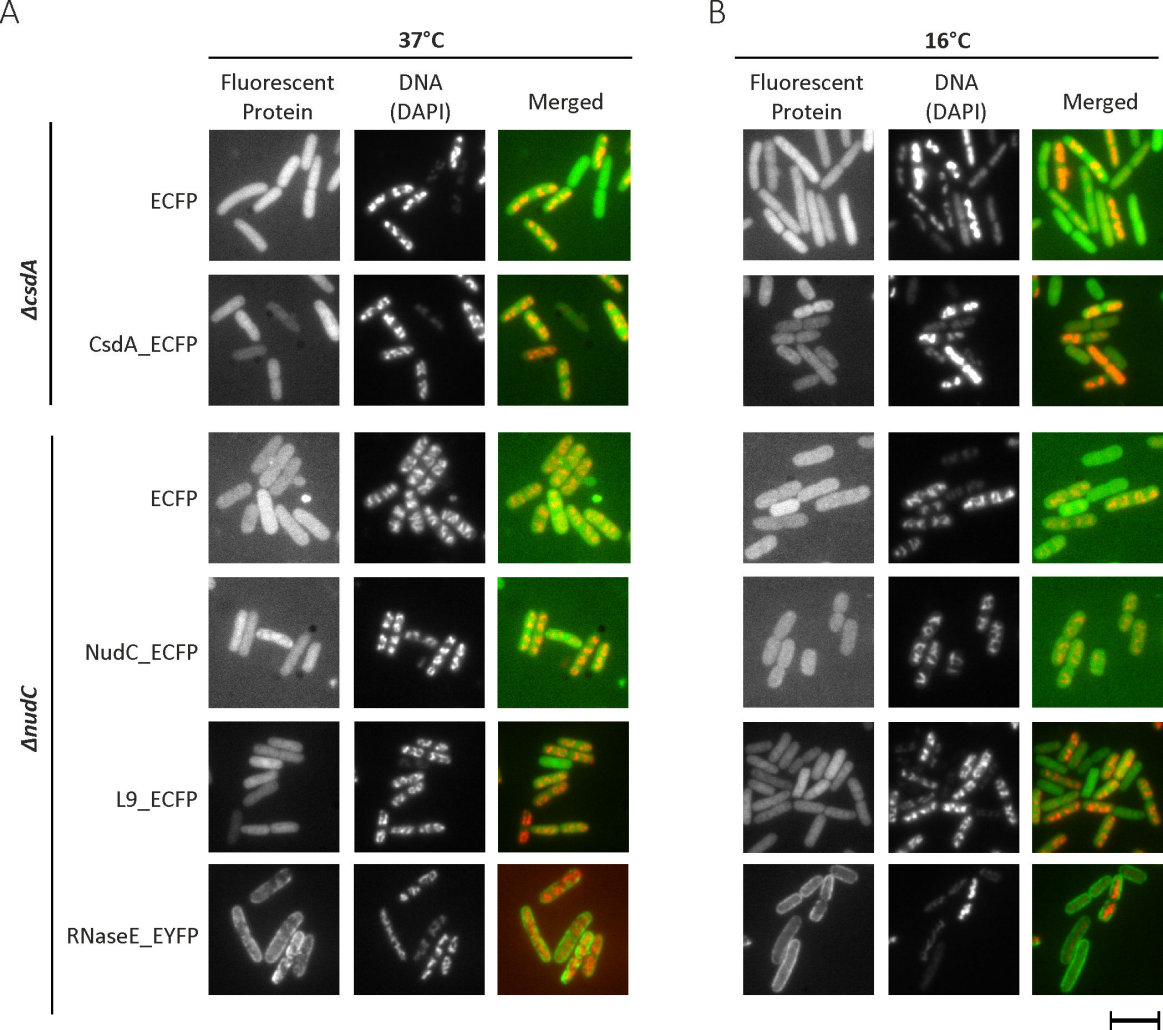
Cellular localization of NudC and CsdA tagged with the ECFP protein. Strains Δ*nudC* and Δ*csdA* deficient in NudC and CsdA, respectively, were complemented with plasmids encoding either fluorescent protein ECFP alone or C-terminal ECFP fused to NudC, CsdA, and ribosomal protein L9. RNA degradosome component RNaseE was fused to EYFP. Following induction with optimized concentrations of arabinose, the subcellular distribution of the fluorescent protein was evaluated either at 37°C (**A**) or after a 30 min shock at 16°C (**B**) using a fluorescence microscope. Nucleoids were stained using a blue-fluorescent DNA-specific dye 4′,6-diamidino-2-phenylindole (DAPI). Representative pictures from two independent replicas are shown. Scale bar: 5 µm.

Protein localization in a living cell can provide additional insights into its function therefore, we examined the cellular distribution of NudC and two of its interactors, CsdA and the large ribosomal subunit protein L9, as well as RNase E (a core scaffold element of the RNA degradosome was used to investigate if the degradosomal association is present for NudC), fused with enhanced cyan or yellow fluorescent proteins (ECFP or EYFP, respectively) (Fig. 4; Fig. S9). Visualization of a fluorescent signal by an upright microscope confirmed that RNase E is tethered to the bacterial inner membrane (Fig. S10). This result agreed well with a recent study showing that degradosomes form membrane-associated punctate bodies (29). On the contrary, ECFP-tagged NudC, CsdA, and L9 have been localized to the cytoplasm, while unfused fluorescent proteins were observed in both the nucleoid and the cytoplasm. Strains expressing the NudC-ECFP and CsdA-ECFP fusion proteins in the Δ*nudC* and Δ*csdA* backgrounds, respectively, could rescue the deletion phenotypes—increased motility of Δ*nudC* cells (this phenotype is described below) and slow growth of Δ*csdA* at low temperatures, indicating the presence of fluorescent protein tags does not disrupt their functionality (Fig. S11). The diffuse cytoplasmic distribution of NudC and CsdA suggests that their functions may not solely rely on the degradosome, if indeed they are associated with this process. Given that NudC and CsdA are not exclusively confined to the degradosome, it is plausible that they have the ability to perform their functions, at least partially, independently of the degradosome.

### Both NudC and CsdA proteins impact NAD modification of RNAs

To determine the effect of NudC and its interaction partner CsdA on RNA modification under cold shock conditions, we compared the global 5′-NAD-RNA levels in wt and mutant strains. The total amount of 5′-NAD-RNA was considerably higher in ∆*nudC* deletion strains at low temperatures, reaffirming the key role of this protein in 5′-NAD level regulation (Fig. S8C). Conversely, ∆*csdA* deletion did not have a substantial effect on the modification levels, suggesting that on a global scale, CsdA is not essential for deNADing. Still, we proposed that NudC could contribute to the cold response by adjusting the activity of CsdA on specific RNAs and/or *vice versa*; CsdA could modulate the stability of NAD modification.

**FIG 5:**
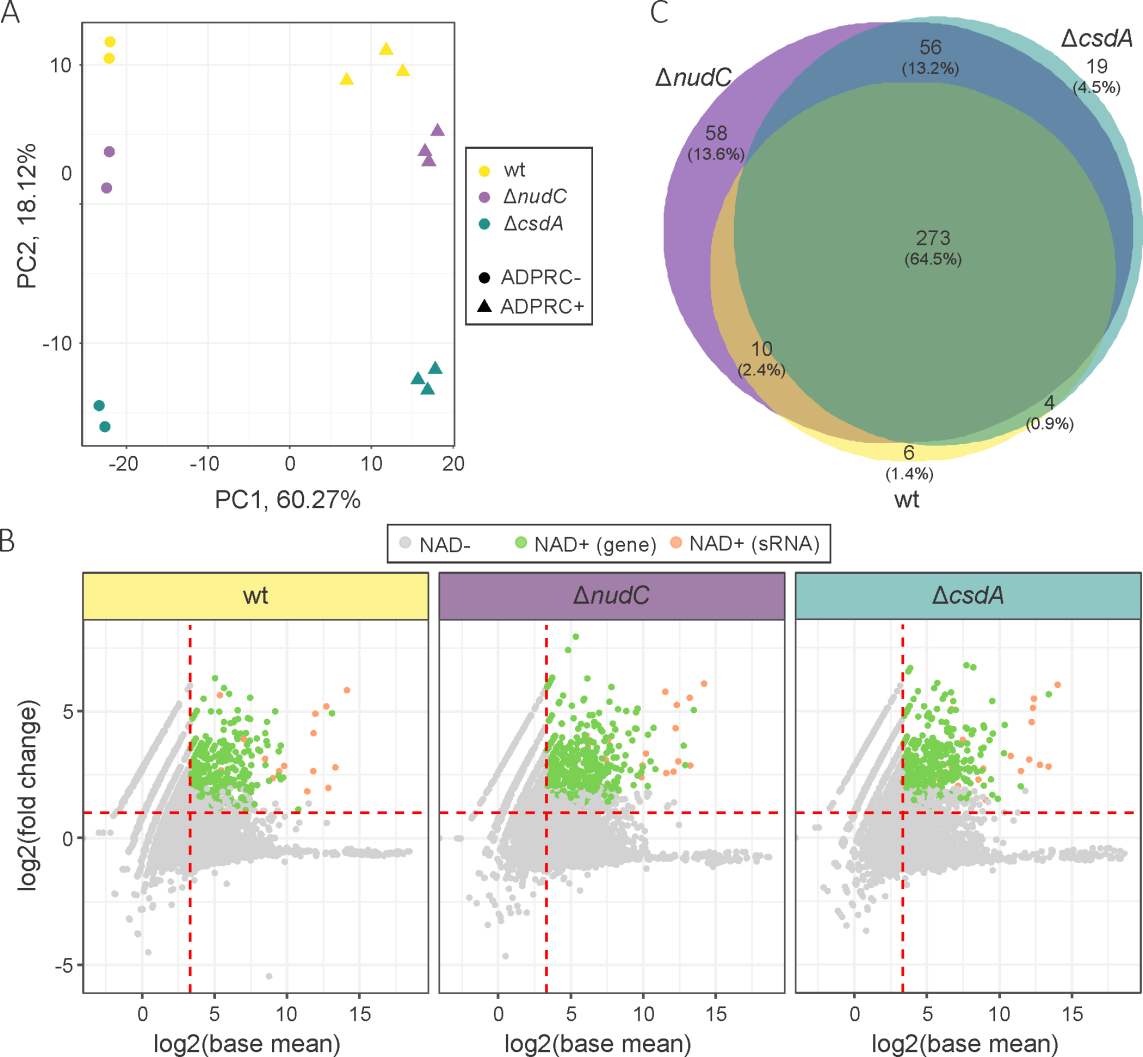
Results of NAD captureSeq. (**A**) Principal component analysis of sequencing data. +ADPRC and −ADPRC samples treated or not with ADP ribosylcyclase in the presence of 3-azido-1-propanol, respectively. (**B**) Scatter plots showing the abundance of significantly enriched 5′-NAD-RNAs in the wt, *∆nudC*, and *∆csdA* strains. Adjusted *P* value <0.05, base mean level of reads ≥10. (**C**) Venn diagram representing the overlap of 5′-NAD-RNAs identified in the wt, *∆nudC*, and *∆csdA* strains. wt, wild-type BW25113; *∆nudC* and *∆csdA, E. coli* mutant strains defective in *nudC* and *csdA* genes, respectively.

To thoroughly assess the outcome of *nudC* and *csdA* depletion on the 5′-RNA modification with NAD at the level of individual transcripts, we performed NAD captureSeq (30) using total RNA extracted from exponential-phase cultures. In +ADPRC samples, ADP ribosylcyclase replaced NAD^+^ moieties with 4-pentyn-1-ol, followed by biotinylation of the alkyn via azide-alkyne cycloaddition; in contrast, in −ADPRC samples, the enzyme was absent. Principal component analysis (PCA) revealed that 5′-NAD-RNA-enriched samples from different strains (+ADPRC) form separate PC2, which accounts for 18% of the variation, clusters and have distinct scores for PC1 (for 60% of the variation) relative to −ADPRC controls (Fig. 5A); 293, 397, and 352 RNA species were considered significantly enriched over the background in the wt, ∆*nudC*, and ∆*csdA*, respectively, after applying a log2-fold change (+ADPRC/−ADPRC) cut-off of ≥1 (to select high-confidence 5′-NAD-RNAs, we added the proviso that a log2-fold change ≥2 must be revealed in at least one of the three strains), an adjusted *P* value <0.05, and a base mean level of reads ≥10 (Fig. 5B and Additional file 2). Altogether, we identified a total of 426 modified RNAs, 95% of which were protein-coding mRNAs and 5% small regulatory RNAs (sRNAs). The majority of 5′-NAD-RNAs (64%) were detected in all samples (Fig. 5C). As expected, 114 (27%) of transcripts undiscovered in wt were present in ∆*nudC*. Surprisingly, approximately half of them (31) were also detected in CsdA-deficient cells. Moreover, 19 of the 5′-NAD-RNAs were only observed in the ∆*csdA* strain, signifying that CsdA, perhaps in conjunction with NudC, can also modulate the NAD epitranscriptomic landscape in *E. coli*.

The compilation of three published data sets (2, 32, 11) yielded a total of 302 5′-NAD-RNAs in several *E. coli* strains grown to exponential or stationary phases. The comparison revealed that the majority of RNA species detected in our work (54% and 41% of all RNAs and sRNAs, respectively) were recognized as being NAD-modified for the first time (Fig. S12). We propose that this different composition may result from dissimilar environmental (cold), mutational (*∆nudC* and *∆csdA*), and genetic (diverse strains) conditions acting on them.

**FIG 6:**
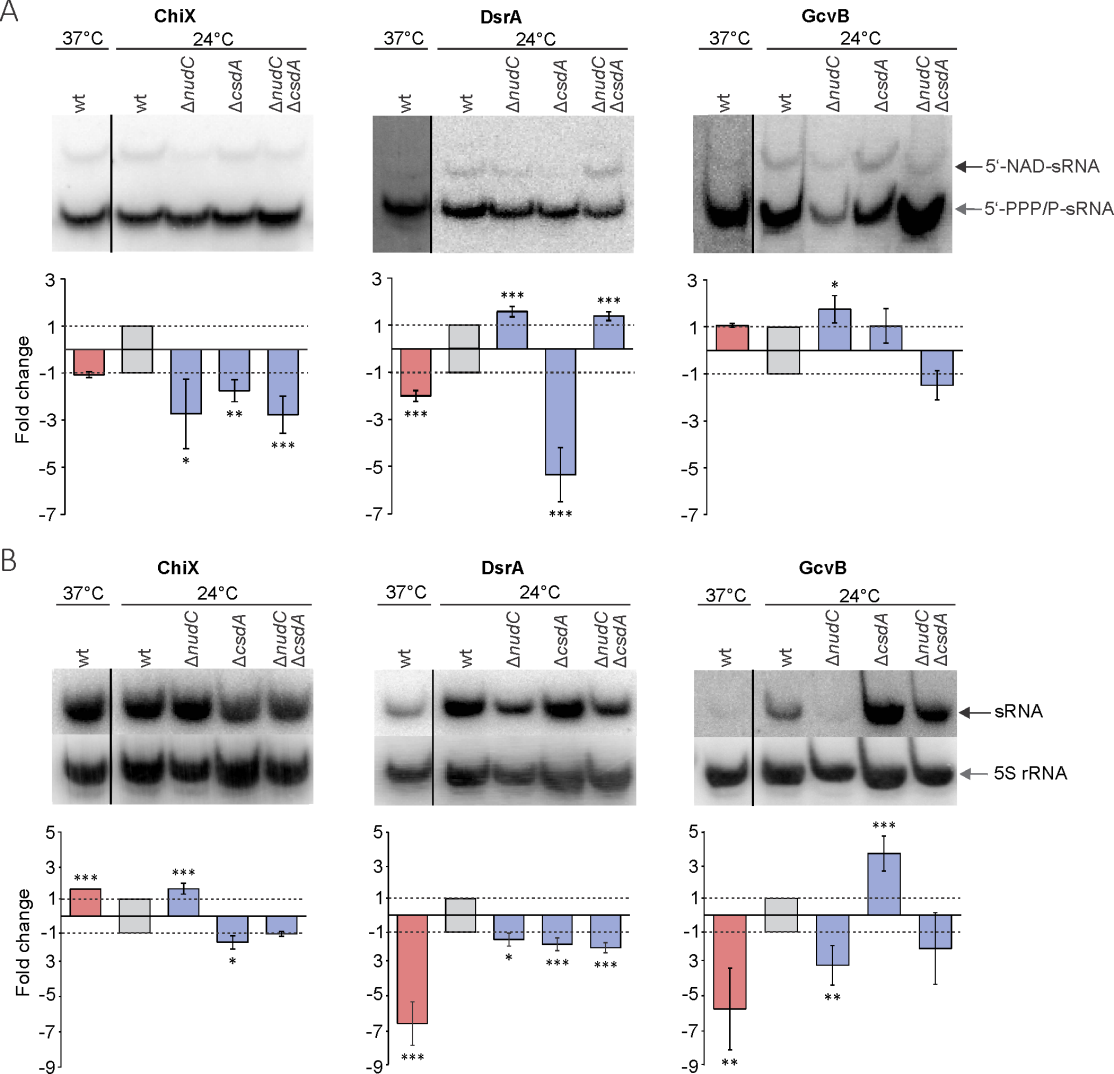
The effect of temperature and NudC or CsdA protein deletion on the 5′-NAD modification level and the amount of particular sRNA. (**A**) Northern blot analysis of three 5′-NAD-capped small RNAs. 5′-NAD-RNA and 5′-PPP/P-RNA were separated on an 8% denaturing PAA gel containing 0.5% APB. The relative amount of 5′-NAD-sRNA was evaluated with respect to the wt strain at 24°C after the correction for 5′-PPP/P-RNA. (**B**) The total amount of sRNA depends on the presence of NudC hydrolase and CsdA helicase. RNA purified from bacteria grown to an early stationary phase was fractionated on a 10% denaturing PAA gel, and the amount of particular sRNA was calculated relative to the wt strain at 24°C after the correction for 5S rRNA loading control. Three independent biological replicates were used to calculate the mean ± SD. The significance of the observed changes as compared to the wt strain at 24°C was calculated using logistic regression analysis. ***P < 0.001, **P < 0.01, *P < 0.05.

The NAD captureSeq method is non-quantitative, and thus the obtained data sets of enrichment values may not be reliable enough to quantify modification changes among strains. Since small RNAs were highlighted as important stress-response regulators (33), we further aimed to compare the abundance and modification level of three selected sRNAs (ChiX, DsrA, and GcvB) isolated from stationary phase BW25113 wt, Δ*nudC*, Δc*sdA*, and double deletion Δ*nudC*Δ*csdA* strains cultivated at 24°C as well as wt strains grown at 37°C (Fig. 6; Table S2) using a Northern blot experiment. To assess the total amount of particular sRNA, the RNA samples subjected to analysis were separated on a conventional denaturing polyacrylamide gel, while acryloylaminophenyl boronic acid (APB) affinity gel electrophoresis (34) enabled the evaluation of the 5′-NAD-RNA/unmodified RNA ratio in the sample (Fig. S13). The blots of wt samples exhibited a clear increase in the proportion of 5′-NAD-RNA at 24°C only for DsrA, indicating that a limited pool of cellular RNAs undergoes a substantial non-canonical capping alteration as a response to the changing environmental conditions (Fig. 6A). We suggest that this particular pool could be responsible for the aforementioned cold-induced increase in the global level of NAD-capped RNA (Fig. 1B). Accordingly, a 2-fold increase in the proportion of 5′-NAD-RNA together with a 6.5-fold change in the RNA amount (Table S2) led to a 13-fold higher cellular concentration of NAD-capped DsrA at 24°C than at 37°C. Noticeably, whereas DsrA synthesis is known to be induced at low temperatures (35), it was demonstrated, for the first time to our knowledge, that GcvB expression is also under temperature control, at least during the entry of bacteria into the stationary phase (5.6-fold higher level when bacteria were grown in cold). The expression of GcvB as well as ChiX at 24°C was affected oppositely by the deletion of *nudC* and *csdA* genes (Fig. 6B). In contrast, the level of these sRNAs in the double mutant was similar to that in the wt strain, indicating that a balance between two proteins may determine the production and/or turnover of certain sRNAs. This crosstalk could potentially take place in the RNA degradome, given previous observations that GcvB is mainly degraded by RNase E (36).

Contrary to expectations of higher yield, the NAD-capping level of ChiX, one of three tested sRNAs, was reduced in the Δ*nudC* strain (Fig. 6A; Table S2). This result suggests that other factors could regulate the modification level of certain RNAs. It is worth noting that in the case of these three NAD-capped sRNAs, the effect of *nudC* deletion on 5′-NAD-RNA and its total level was of the opposite sign, while the lack of CsdA protein had a similar impact on both subsets of RNA. To our surprise, the inactivation of *csdA* also caused a decrease in 5′-NAD-RNA levels, especially substantial in the case of DsrA (more than 5-fold). In double-knockout cells, the DsrA modification recurred to the level of the Δ*nudC* strain (Fig. 6A), despite minor variation in intracellular RNA concentration (Fig. 6B). Consequently, CsdA may act to protect this particular modified sRNA from (i) degradation or (ii) decapping by NudC. Still, further examination is needed to properly assess the CsdA effects on specific sRNAs.

### NudC hydrolase affects the bacterial transcriptome landscape

**FIG 7:**
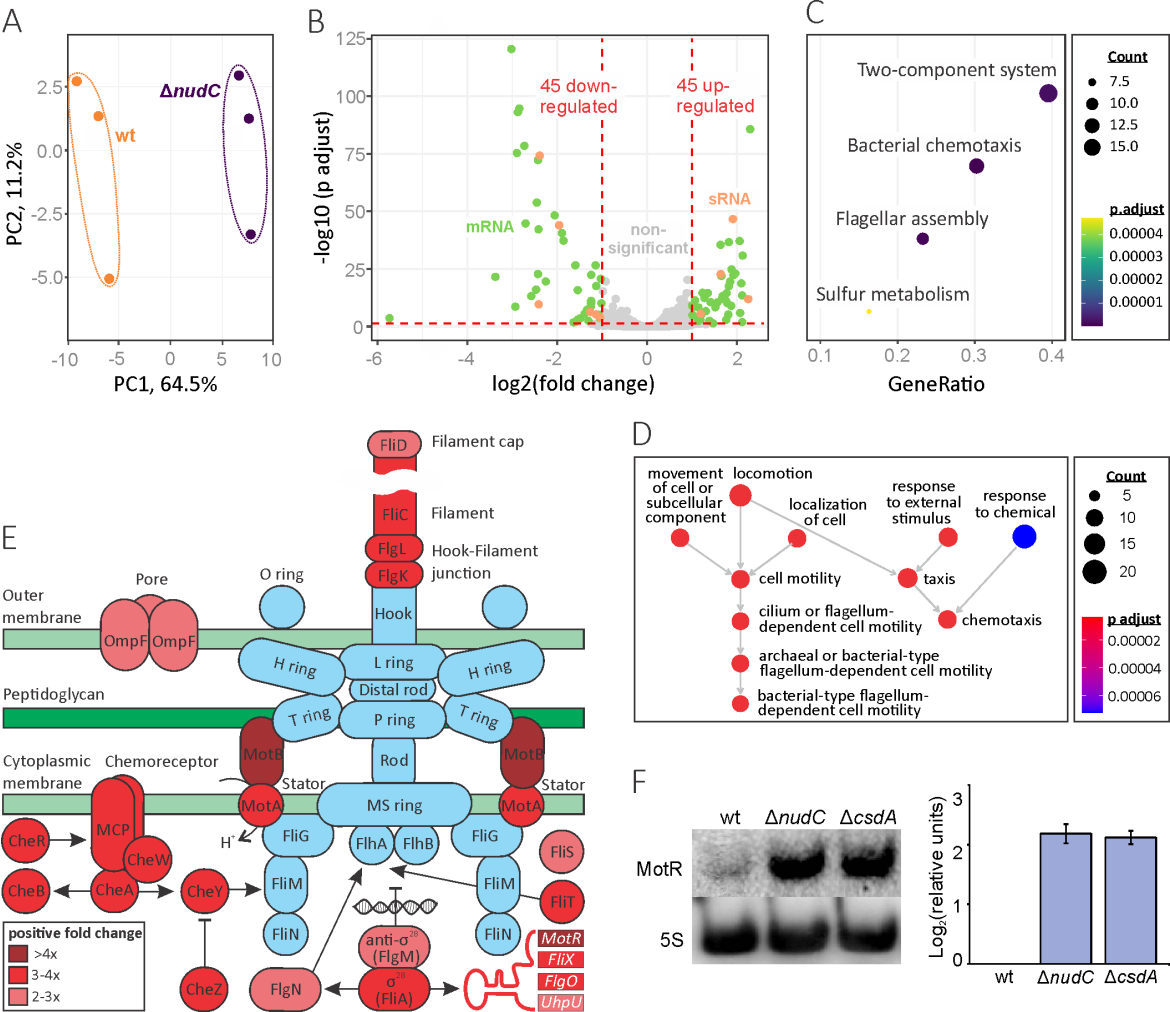
The effect of Δ*nudC* knockout on gene expression. (**A**) PCA analysis of wild-type *E. coli* BW25113 (wt) and Δ*nudC* RNA-seq data sets. (**B**) The Volcano plot of differentially expressed transcripts in the Δ*nudC* strain. Significantly dysregulated mRNAs and sRNAs that passed the ≥1 log2-fold change and <0.05 adjusted *P* value thresholds are shown in green and orange, respectively. (**C**) KEGG pathway functional classification of the differentially expressed transcripts. (**D**) The molecular function category of the GO classification. (**E**) Affected genes on a flagellar model. Proteins and sRNAs (MotR, FliX, FlgO, UhpU) are represented by ovals and rectangles, respectively. The red color depicts the up-regulated genes in the Δ*nudC* strain. (**F**) MotR sRNA expression increases in both Δ*nudC* and Δ*csdA* strains (4.5 ± 0.5- and 4.3 ± 0.3-fold, respectively). 5S rRNA was used as a quantitative control. Three independent biological replicates were used to calculate the mean ± SD.

To determine the underlying RNA abundance changes induced by NudC hydrolase, we carried out whole RNA sequencing of wt and Δ*nudC* bacteria grown at 24°C until the exponential phase, where cell division proceeds at a constant rate. The PCA plot of data sets showed a clear separation of the wt and mutant samples (Fig. 7A), suggesting that NudC might modulate the RNA pool during cold-stress conditions. The comparative transcriptomic analysis, using a cut-off criteria base-2 log fold-change threshold of ≥1 and an adjusted *P* value below 0.05, revealed significant differential expression of 90 transcripts in the Δ*nudC* strain, of which an equal number (45) were up- and down-regulated (Fig. 7B and Additional file 3). We identified 78 protein-coding genes, 10 sRNA genes, and 2 pseudogenes. Notably, *nudC* knockout does not affect the transcription of *csdA*. To further evaluate hydrolase-mediated transcriptomic changes, Kyoto Encyclopedia of Genes and Genomes (KEGG) and Gene Ontology (GO) analyses were used. KEGG pathway enrichment of differentially expressed genes revealed four categories, three of which—bacterial chemotaxis, flagellar assembly, and two-component system—contain overlapping elements (Fig. 7C; Fig. S14A). Consistently, GO functional classification revealed two dominant groups among the biological process terms associated with cell movement in response to stimulus (Fig. 7D; Fig. S14B and C). Interestingly, the majority of genes controlled by the alternate sigma transcription factor σ^28^, also known as FliA or RpoF, were up-regulated as a result of *nudC* inactivation (Fig. 7E; Fig. S15). This factor coordinates class III flagellar genes (late time of activation) involved in the synthesis of the chemotaxis system and complete flagellum (37). Furthermore, all four σ^28^-dependent sRNAs regulating the timing of flagella biosynthesis (MotR, FliX, FlgO, UhpU) were differentially expressed in our experiment (38). In contrast, only two class II operons (middle time of activation)*—fliAZY* and *flgAMN*, encoding sigma factor σ^28^ and anti-sigma factor FlgM, were significantly up-regulated in the Δ*nudC* strain. Still, albeit to a lesser extent (1.7–1.8-fold change), the expression of positive *flhDC* regulator McaS and negative sRNA regulators OmrA and OmrB (39, 40) was up- and down-regulated, respectively, leading to a statistically significant 1.9-fold increase in the amount of mRNA of flagellar transcriptional regulator *flhC*. Thus, the transcription of specific class II operons in the Δ*nudC* strain may also be facilitated by the FlhDC flagellar transcription regulator.

Northern blot analysis performed using RNA from exponentially growing cultures at 24°C not only reaffirmed an approximately 5-fold augment in *MotR* level in the absence of NudC but also revealed that the lack of functional *csdA* affects the cellular abundance of this Hfq-bound small RNA controlling flagella synthesis and motility (38) (Fig. 7F). On the contrary, we observed a sharp decrease in MotR expression (−21.8 ± 11.3-fold) following the introduction of the NudC-coding plasmid into the cell (Fig. S16A). Although the expression of inactive NudC(E178Q) also impacted the abundance of MotR, the inhibition was not as pronounced (−4.9 ± 4.6-fold). This suggests that catalytic activity, at least to some extent, is necessary for the regulation of this sRNA. This information indicates that NudC and perhaps CsdA could jointly contribute to bacterial movement.

### Effects of NudC and CsdA on bacterial motility

**FIG 8:**
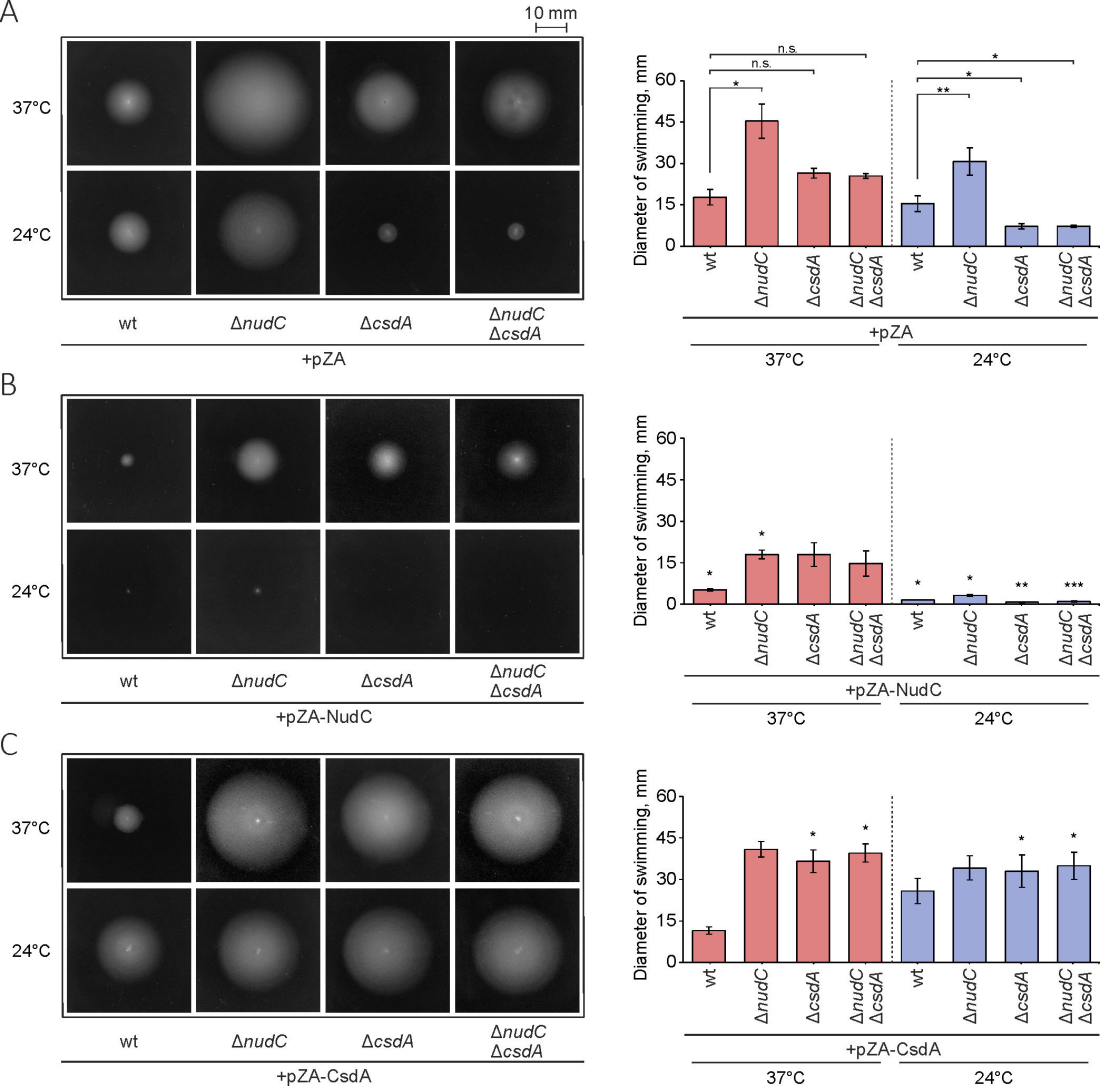
Swimming phenotype of wild-type BW25113 and its derivatives. (**A**) Images of a colony spreading of strains with an empty pZA vector. The effect of plasmid-mediated expression of *nudC* (**B**) and *csdA* fused with *ecfp* (**C**) on bacterial motility. Cells were spotted on soft agar (0.25%) with IPTG and incubated for 16 and 20 h at 37°C and 24°C, respectively. The mean swimming diameter ± SD for each strain was calculated from three biological replicates. *P < 0.05, **P < 0.01, and ***P < 0.001 denote statistical significance as determined using student’s *t*-test. Lines with asterisks indicate a significant difference between the connected bars. Asterisks positioned above bars in the middle and lower plots indicate a significant difference between the corresponding strain with an empty pZA vector. "n.s." denotes non-significant results. See Table S3.

Bacterial motility is often altered as an adaptive response to diverse environmental challenges (41, 42). Our results of the comparative transcriptomic analysis showed that the deletion of *nudC* promotes the expression of flagellar components in *E. coli*. Moreover, the universal stress protein UspG, which was identified as one of the NudC-interacting factors, is important for bacterial adhesion and motility (27). With this in mind, we sought to establish the regulatory effects of both NudC and its interaction partner CsdA on the swimming speed of *E. coli* cells. Analysis of the Δ*nudC*, Δ*csdA*, and Δ*nudC*Δ*csdA* strains revealed that only cells without the NudC displayed enhanced motility compared to the wt and Δ*csdA* strains at both normal and low temperatures (Fig. 8A; Table S3). At the same time, this swimming phenotype is not caused merely by the growth defects because Δ*nudC* and wt have similar growth curves (Fig. S17). Remarkably, the Δ*nudC*Δ*csdA* double-deletion strain was devoid of the fastest swimming capacity, suggesting that the intercommunion between NudC and CsdA may play a role in modulating the phenotypic response. Moreover, while the *csdA* mutation slightly slowed down the bacterial growth at 37°C, similar double-mutant and wt growth curves (except for an extended adaptation period in the mutant) indicate that *nudC* inactivation may attenuate the defect of Δ*csdA* at a convenient temperature. Although Δ*csdA* cells spread poorly compared to wt bacteria at 24°C, we propose that this impairment may be caused by a significant reduction in the overall growth of the CsdA-lacking strains at a lower temperature (Fig. S17B) (28). The expression of plasmid-borne *nudC* retarded cell motility in the tested strains, indicating that the deficiency of NudC specifically led to the aberrant Δ*nud*C phenotype (Fig. 8B). To ascertain whether the changes in motility are attributable to the catalytic activity of NudC, we expressed the NudC(E178Q) mutant protein into the Δ*nudC* strain (Fig. S16B). In agreement with the previously demonstrated results for the motility-regulating MotR sRNA (Fig. S16A), the introduction of the catalytically inactive mutant showed intermediate outcomes in terms of movement reduction. Taken together, these results suggest that the changes in motility could be a result of both the catalytic and non-catalytic activities of NudC. In contrast to *nudC*, the expression of *csdA* significantly improved motility in the majority of strains (Fig. 8C; Table S3). Compared to cells with the pZA vector, lower spread area was only observed for wt cells with pZA-CsdA at 37°C, suggesting that bacterial motility depends on temperature and genetic context. Previous studies have shown that alternative cold shock-responsive factors significantly transform cellular RNomic and proteomic landscapes to adapt to low temperatures (43) when *csdA* expression is highly induced (44). Therefore, we suppose that additive modulators of CsdA or NudC activity may be expressed only under specific conditions. The growth analysis revealed no significant differences between strains and confirmed the functional activity of the pZA-CsdA plasmid that rescued the impaired growth of the Δ*csdA* strain at 24°C (Fig. S17).

Transmission electron microscopy was applied to assess whether the NudC-dependent motility failures depend on flagella function (Fig. S18). Our data show that the Δ*nudC* cells exhibit hyper-flagellation compared to wt cells. Yet, complementation of Δ*nudC* with the plasmid-based *nudC* gene restored the low flagellar density. Thus, we concluded that the interplay between NudC and CsdA affects factors regulating flagellar density and motility in *E. coli*.

Based on our data, we proposed a model explaining the functional relationship between NudC and CsdA in *E. coli* motility, according to which, free or in complex with an unknown protein (e.g., a potential interaction partner, UspG), NudC triggers a decrease in bacterial motility, while CsdA promotes it. The effect of both proteins may be neutralized after the formation of the NudC-CsdA complex (Fig. S19). In conclusion, we have established a hitherto unknown link between cellular motility and the RNA-demodifying enzyme.

## DISCUSSION

In this work, we demonstrate, for the first time, that a larger portion of RNA in *E. coli* bears the 5′-NAD^+^ modification when grown at lower temperatures. This phenomenon persists also during the stationary growth phase at 37°C and 24°C, despite the similar concentrations of intracellular NAD^+^ cofactor. Our data highlight RNA capping as a selective process functioning as one of the stress response pathways in bacterial adaptation. These results add to the accumulating evidence that links 5′-terminal RNA modification to environmental stimuli, such as disulfide stress (7), hormonal influence (5), growth medium (19, 45), and growth phase (3, 8, 9, 11). Our results emphasize the importance of epitranscriptomic information in a broader sense that reaches beyond genome-coded elements, including promoter sequences (3, 8, 9, 19, 32, 46 – 48). In bacteria, higher RNA modification under particular stress conditions could come with the advantage of increased RNA stability (3, 7, 8) and maybe even guarantee the synthesis of specific RNAs critical under certain environmental circumstances (11) as transcription initiation with NAD^+^ is shown to stimulate the promoter escape (46). Indeed, despite the global increase in the total amount of 5′-NAD-RNA at 24°C, we demonstrate that the modified fraction does respond to the temperature change for just one out of three RNAs analyzed using APB affinity gel electrophoresis. It is DsrA, the sRNA, that stimulates the translation of the stress σ factor RpoS (49), which shows a significant increase in the 5′-NAD-RNA portion and total amount. However, the direction of causal dependence between an increase in NAD modification and total transcription as detected in this publication and earlier work (11) awaits further research, as does the effect of 5′-NAD-sRNA on their target genes (47).

The observation that many potential NudC-interacting proteins, including the most prominent one, CsdA helicase, are related to bacterial stress-response pathways further strengthens the link between epitranscriptomic modification and environmental stimuli. Meanwhile, the proteins associated with the RNA degradosome, again including CsdA, seem to connect NAD-cap decapping with RNA hydrolysis, which is critical for bacterial adaptation. This appears to be particularly important, knowing that NudC has no specific RNA-binding activity (23). We can speculate that the interaction between NudC and the RNA chaperone Hfq additionally directs hydrolase toward Hfq-bound sRNAs, the repertoire of which bears NAD modification (2, 11, 32) and needs fast alterations in an ever-changing environment (50). On the other hand, as the majority of NudC and CsdA proteins are localized throughout the cytoplasm, functions of these proteins must reach far beyond the membrane-anchored RNA degradosome (29). Specifically, NudC associates with ribosome proteins. Along with the fact that both interaction partners, CsdA and Hfq, contribute to ribosome biogenesis and assembly (51 – 53), it supports the notion of the possible involvement of the decapping protein in bacterial translation.

As we provide evidence for direct interaction between NudC and CsdA, it is important to note that other Nudix hydrolases also function in complexes with additional proteins. Indeed, the association of a Nudix family pyrophosphatase with a cold shock DEAD-box RNA helicase, RhlE, has been observed in the aquatic Gram-negative bacterium *Caulobacter crescentus* (54), indicating that cooperative helicase-hydrolase relationships in stress responses may be common in bacteria. *E. coli* 5′-PPP-RNA hydrolase RppH is activated thereupon, forming a heterotetrameric complex with diaminopimelate epimerase DapF (55, 56). The activity of the eukaryotic m^7^G decapping protein Dcp2 is stimulated by Dcp1, interacting through their N-terminal regions (31), similar to NudC and CsdA. We demonstrate that both interacting partners localize in bacterial cytoplasm and their deletions show functional overlap in bacterial motility. Unexpectedly, we discover that the Δ*nudC* increases the swimming speed of *E. coli*, while the absence of CsdA appears to have the opposite effect. To our knowledge, at least two other Nudix enzymes have been shown to regulate bacterial motility, though in the reverse direction. The deletion of *E. coli* Np_n_N-cap hydrolase *apaH* (7, 9) results in a loss of bacterial motility and decreased flagellin synthesis (57), while the plant pathogen *Pseudomonas syringae*, deficient in NudC accumulation, displays severely impaired swimming and swarming abilities (58).

Out of all phenotypes described in NudC*-* and CsdA*-*lacking strains, the most striking one appears to be the effect of both proteins on the multiplicity of NAD-modified RNA species. We show that despite CsdA’s inability to globally regulate the cellular level of RNA NAD capping, a total of 75 5′-NAD-RNA not found in wt have been elucidated in the Δ*csdA* strain, covering almost one-third of the species specific to Δ*nudC* (56 out of 198). Moreover, CsdA affects two out of three NAD-capped sRNAs analyzed in APB gels. CsdA itself does not display hydrolase activity, but it could act as a mediator between NudC and the degradosome. On the other hand, we demonstrated that NudC can both reduce and even increase the amount of NAD-sRNA, depending on the nature of the particular molecules. Although the latter effect contradicts the main enzymatic function of NudC hydrolase (23), an even more drastic one was detected in the deletion strain of the yeast homolog of NudC Npy1. In that particular case, while the number of unique NAD-capped RNAs doubled, the NAD-modification ratio increased only for 164 out of 1,177 RNAs (48). What is more, we revealed that NudC functions as the regulator of not only NAD-modified RNAs but also of their total amount. Similarly, the lack of BsRppH in *B. subtilis* (3), Npy1 in yeast BY4742 (48), and DXO1 in *Arabidopsis thaliana* is known to affect the expression of hundreds of genes (5, 59, 60). In the future, it would be interesting to find out whether the hydrolase activity of bacterial NudC is necessary to cause extensive alterations in transcriptome profiles. A recent study of DXO1, a member of the DXO/Rai1 family in *Arabidopsis*, revealed a non-enzymatic function of this deNADing protein: the activation of RNA guanosine-7 methyltransferase responsible for the methylation of the guanosine cap (61). Together, the data obtained point to both the connection among all forms of RNA as well as the biological functions of 5′-NAD-RNA hydrolases extending far beyond the RNA decapping process.

## MATERIALS AND METHODS

### HPLC-MS/MS analysis of 5′-NAD^+^-RNA

For HPLC-MS/MS analysis, bacterial cultures were grown in LB medium at 37°C or 24°C and collected at specific growth stages after premixing with 1/8 vol of ice-cold STOP solution (5% acid phenol, 95% ethanol). Total RNA was extracted using RNazol RT (RN 190) (Molecular Research Center, Inc.) according to the manufacturer’s recommendations, additionally purified using the RNA Clean & Concentrator-100 Kit (Zymo Research), treated with TURBO DNAse (Invitrogen), and further recovered with the RNA Clean & Concentrator-100 Kit. After splitting into two, half of the sample containing 2.5 µg/µL RNA was treated with 0.05 U/µL P1 nuclease (Sigma Aldrich) for 16 h at 37°C, while the other half was incubated at the same conditions only without the P1 nuclease. The digestion products were purified with a Microcon-10 kDa filter (Millipore) and analyzed by an integrated HPLC/ESI-MS/MS system (Agilent 1290 Infinity/6410B triple quadruple) after loading on a Discovery SH C18 column (Supelco), followed by elution with a linear gradient from 0.02% formic acid in water to 100% acetonitrile. The mass spectrometer was operating in a positive ion mode, and the intensity of modification-specific ion transitions was recorded (*m*/*z* 664.1–542.1). The amount of detected NAD^+^ was evaluated using MassHunter Qualitative Analysis software (Agilent Technologies), normalized according to P1-untreated samples, and quantified based on a calibration curve. All results were calculated from three biological replicates, and their significance was evaluated using the Welch *t*-test and pairwise comparisons.

### Affinity-tag pull-down

For affinity-tag pull-down experiments, *E. coli* BL21(DE3)RIL was transformed with a plasmid containing the *nudC* gene with six histidine residues, pET28a-NudC-His, or a parental vector without the insert, pET28a. The construction of plasmids used in affinity-tag pull-down experiments is detailed in supplementary methods. Selected colonies were inoculated into LB medium with appropriate antibiotics and grown at 37°C and 200 rpm. Overnight bacterial cultures were diluted 100-fold into fresh LB medium supplemented with required antibiotics and grown at 37°C until OD_600_ 0.6–0.8. After induction with 0.1 mM IPTG, bacteria were further grown at 37°C for 4 h or at 16°C for 18 h with shaking at 200 rpm. Bacteria were collected by centrifugation for 15 min at 3,000 × g at 4°C, and pellets were washed with water. For experiments with the BW25113 strain, no plasmid, antibiotic, or inducer was used.

For bacterium lysis, pellets were resuspended in lysis buffer A (10% saccharose, 50 mM Na_2_HPO_4_ × 2H_2_O, 100 mM NaCl, Complete EDTA-free protease inhibitor cocktail tablets (Roche), 0.1% Triton X-100, 0.1 µM pepstatin, pH 7.4) and sonicated with a Vibra cell ultrasonic processor for 6 min, using 1 s pulses and 60% amplitude. The lysates were centrifuged for 20 min at 20,000 × g 4°C, and the supernatants were collected and filtered through a 0.45 µm filter.

Chelating Sepharose Fast Flow sorbent (GE Healthcare) was prepared by removing ethanol and washing two times with an equal volume of autoclaved water, one time with 0.1 M NiSO_4_, and then again five times with autoclaved water. Each time, the supernatant was removed by centrifuging the sorbent for 5 min at 500 × g 4°C. Finally, the sorbent was mixed with an equal volume of lysis buffer B (10% saccharose, 50 mM Na_2_HPO_4_ × 2H_2_O, 100 mM NaCl, pH 7.4).

All further steps were performed at 4°C with three different types of samples: (i) 5 mg of bacteria lysate with expressed NudC-His, (ii) 5 mg of bacteria lysate without, or (iii) with 243 µg of recombinant NudC-His. All three samples were mixed with previously prepared sorbent at a ratio of 8.3:1 and incubated for 1 h 30 min at 8°C. Where indicated, 10 µg of RNase A (Thermo Fisher Scientific) was added during this step. After incubation, the supernatant was discarded by centrifuging the samples for 5 min at 500 × g. Proteins that interact with the sorbent non-specifically were removed by incubating samples with 5 vol of 60 mM imidazole containing lysis buffer B for 5 min and then centrifuging for another 5 min at 500 × g. In total, the previous step was repeated five times. Sorbent beads were incubated with 8 M urea for 10 min at room temperature to remove only co-purified proteins, thus minimizing NudC-His quantity for MS analysis. Alternatively, all the sorbent-bound proteins were separated from the sorbent by heating the samples for 5 min at 95°C and analyzed by SDS-PAGE.

### LC-MS^E^-based protein identification

For band-specific analysis, selected protein bands were cut from SDS-PAGE using a sterile razor blade, sliced into 1–2 mm^3^ pieces, and incubated twice with 200 µL of destaining solution (25 mM NH_4_HCO_3_, 50% CH_3_CN) for 30 min at 37°C. Then, proteins were reduced with 50 mM TCEP in 25 mM NH_4_HCO_3_ for 10 min at 60°C. Cysteine residues were alkylated with freshly prepared 100 mM iodoacetamide in 25 mM NH_4_HCO_3_ for 1 h at room temperature. Samples were washed twice with destaining solution for 15 min at 37°C, dehydrated by incubating with CH_3_CN for 15 min at room temperature, and digested overnight by trypsin at 37°C. Peptides were then collected by centrifugation and further extracted from the gel after incubation with 5% CF_3_COOH and 50% CH_3_CN for 1 h at 37°C.

For the proteome-wide screen, samples were processed in 0.5 mL Amicon Ultra 30k filtration units according to the FASP protocol (62). In brief, each sample was diluted to a volume of 200 µL using freshly prepared urea buffer (8 M urea in 0.1 M Tris–HCl, pH 8.5). Proteins were alkylated with 50 mM iodoacetamide and washed twice with urea and twice with 50 mM NH_4_HCO_3_. Proteins were digested overnight by trypsin at 37°C. Peptides were then collected from a filter by centrifugation, which was further washed with 40 µL 20% CH_3_CN. The eluates were acidified with 10% CF_3_COOH and, together with peptides prepared for band-specific analysis, lyophilized in a vacuum centrifuge. The lyophilized peptides were redissolved in 0.1% formic acid.

The peptide mixture was separated on the Waters Acquity ultra-performance LC system (Waters Corporation, Wilmslow, UK) using an HSS T3 250 mm analytical column at a flow rate of 300 nL/min. LC-(UD)MS^E^ (63) data were acquired in positive ion and resolution mode with SYNAPT G2 using Masslynx (version 4.1) in alternating low energy (MS), coupled with ion mobility separation (IMS), and high energy (MS^E^) modes with a mass scan range from 50 to 2,000 *m*/*z* and scan time set to 0.8 s.

The raw data were processed with the ProteinLynx Global Server (PLGS) version 3.0.1 (Waters Corporation, UK). Raw data were lock mass-corrected using the doubly charged ion of [Glu1]-fibrinopeptide B (*m*/*z* 785.8426) and a 0.25 Da tolerance window. Peak lists were generated using the following parameters: (i) low energy threshold was set to 150 counts; (ii) elevated energy threshold was set to 50 counts; and (iii) intensity threshold was set to 750 counts. Database searching was performed with the PLGS search engine using automatic peptide tolerance and fragment tolerance, with minimum fragment ion matches of one per peptide and three per protein, false discovery rate (FDR) <4%. Trypsin as the cleavage protease was used for data analysis; one missed cleavage was allowed; the fixed modification was set to carbamidomethylation of cysteines; and the variable modification was set to oxidation of methionine. The Uniprot *Escherichia coli* (strain B/BL21-DE3) database downloaded on 12 February 2020 was used for protein identification.

Protein quantification was calculated using the Hi3 method (64) based on the 50 fmol loading of trypsin-digested phosphorylase b (SwissProt P00489).

### BATCH assay

For the analysis of NudC and CsdA protein interactions *in vivo*, the *E. coli* BTH101 strain was chemically transformed with plasmids containing either NudC- or CsdA-encoding genes fused to T18 or T25 adenylate cyclase subunits (65). In total, eight different combinations were made: pUT18C-CsdA/pKNT25-NudC, pUT18C-CsdA/pKT25-NudC, pUT18-CsdA/pKNT25-NudC, pUT18-CsdA/pKT25-NudC, pUT18C-NudC/pKNT25-CsdA, pUT18C-NudC/pKT25-CsdA, pUT18-NudC/pKNT25-CsdA, and pUT18-NudC/pKT25-CsdA. As a control, the *E. coli* BTH101 strain was transformed with plasmids containing just the subunits of adenylate cyclase: pUT18C/pKNT25, pUT18C/pKT25, pUT18/pKNT25, and pUT18/pKT25.

To determine which region of each protein is responsible for the interaction between NudC and CsdA, the BTH101 strain was chemically transformed with another set of plasmid pairs: pKNT25-NudC/pUT18C-CsdA, pKNT25-NudC-N/pUT18C-CsdA-N, pKNT25-NudC-N/pUT18C-CsdA-C, pKNT25-NudC-C/pUT18C-CsdA-N, pKNT25-NudC-C/pUT18C-CsdA-C, pKNT25-NudC/pUT18C-CsdA-N, pKNT25-NudC/pUT18C-CsdA-C, pUT18C-CsdA/pKNT25-NudC-N, and pUT18C-CsdA/pKNT25-NudC-C. As a control, the *E. coli* strain BTH101 was transformed with plasmids that contained only adenylate cyclase subunits (pKNT25/pUT18C), one of the complex-forming proteins and adenylate cyclase subunits (pKNT25-NudC/pUT18C, pUT18C-CsdA/pKNT25) or only a truncated variant of one of the complex-forming proteins and adenylate cyclase subunits (pKNT25-NudC-N/pUT18C, pKNT25-NudC-C/pUT18C, pKNT25/pUT18C-CsdA-C, pKNT25/pUT18C-CsdA-N).

In all transformations, 0.2 µg of each plasmid was used. One microliter of overnight cultures (grown at 37°C) was placed on LB agar plates containing X-gal (40 µg/mL), IPTG (0.5 mM), and appropriate antibiotics and grown at 30°C. Changes in the color of the colonies were registered after 2 days.

For analysis of NudC interaction with Hfq protein, the set of Hfq-encoding gene plasmids fused to T18 or T25 adenylate cyclase subunit was co-transformed with NudC variants. The construction of plasmids used in BATCH assays is detailed in supplementary methods.

### Fluorescence microscopy

Overnight bacterial cultures were diluted 100-fold with fresh LB media supplemented with an inducer (arabinose and/or IPTG) and incubated to reach midlog (OD_600_ ~0.5). Then, 1 mL of the bacterial suspension was washed three times in PBS buffer and fixed with 4% formaldehyde for 5 min in the dark. The bacteria were washed three times with PBS to remove the remaining formaldehyde before being concentrated to 50 µL and subjected to fluorescence microscopy. In the case of cold shock, 1 mL of the bacterial cultures grown to midlog was incubated at 16°C for 30 min before being fixed with 4% formaldehyde and stained with 1 µg/mL (final concentration) DAPI for 5 min. For microscopy, 3 µL of cell suspension was loaded on a slide and visualized at 1,000 × magnification using a WIBA filter for ECFP/EYFP (460–490 nm excitation and 515–550 nm emission) and a MWU filter for DAPI (330–385 nm excitation and >420 nm emission) on a fluorescence microscope Olympus AX70 with a 100×/1.35 oil immersion lens and a CCD camera Orca (Hamamatsu). The experiment was repeated twice. The images obtained at different wavelengths were merged into a single image using Fiji software.

### RNA library preparation and sequencing data analysis

For RNA library preparation, the RNA was purified as described above from the exponential phase bacterial cultures grown in LB medium at 24°C. The quality of extracted RNA was evaluated using the Agilent RNA 6000 Pico Kit (Agilent Technologies).

NAD captureSeq libraries were prepared according to the modified protocol provided by (30). To be precise, after DNA removal, fragmentation (which was prolonged to 3 min), and ADPRC reaction, RNA was purified using the RNA Clean & Concentrator-100 Kit (Zymo Research). For the click reaction, the RNA was purified and precipitated according to the RNA Clean & Concentrator-100 Kit protocol and incubated with 1 mM of CuBr and 9 mM of THPTA. Then, 70–80 µg of biotinylated RNA was mixed with 25 µL of prewashed magnetic beads, Dynabeads MyOne Streptavidin C1 (Invitrogen), and further processed as described in (30). Following the RNA capture, the magnetic beads were washed four times with Streptavidin wash buffer containing 0.25% Triton and then three times with H_2_O. The RNA 3′-adaptor, containing an 11 nt UMI sequence, was preadenylated using the 5′ DNA Adenylation Kit (New England BioLabs), and 13 PCR cycles were performed.

For the total RNA library, rRNAs were depleted using the RiboMinus Bacteria 2.0 Transcriptome Isolation Kit (Invitrogen). The remaining RNA was incubated for 2 min at 75°C in the RNA fragmentation buffer containing 10 mM Tris-HCl (pH 7.4) and 10 mM ZnCl_2._ The obtained 100–400 nt fragments were dephosphorylated using T4 PNK (Thermo Fisher Scientific) in buffer B for 1 h at 37°C. The RNA library was prepared from 140 ng of fragmented and dephosphorylated RNA according to the NEXTflex Small RNA-seq Kit v3 protocol (PerkinElmer) with the following modifications: the incubation time at 42°C and 72°C was extended to 60 min (steps E and G of the manual); the fragments were amplified for 9 PCR cycles (step G); and steps F and H1 were performed without size selection as described in the alternative library prep protocol. RNA libraries up to 500 nt in length were purified from PAA gel as outlined in step H2.

The size distribution of prepared libraries was evaluated by capillary electrophoresis (Agilent High Sensitivity DNA Kit, Agilent Technologies) and quantified by the KAPA Library Quantification Kit (Roche). Deep bi-directional sequencing of NGS libraries was performed at EMBL Genomics Core Facilities using NextSeq 1000/2000 P2 Reagents (100 Cycles) v3 (Illumina).

The quality of raw sequencing reads was evaluated using the FASTQC program v0.11.9 (https://www.bioinformatics.babraham.ac.uk/projects/fastqc/). RNA-seq raw reads were quality (PHRED threshold 20) and length (minimum length 20) filtered using Trim Galore! v0.6.5 (https://www.bioinformatics.babraham.ac.uk/projects/trim_galore/). NAD captureSeq raw reads were trimmed with SeqPurge (66) (adapter removal and length filtering) and cutadapt (67) (poliG/poliA removal). Processed reads were mapped to the reference *E. coli* BW25113 strain K-12 (NZ_CP009273.1) genome using hisat2 v2.2.0 (68) (options --no-unal --no-spliced-alignment -X 500 --no-mixed --no-discordant for the RNA-seq data and --score-min L,0,–0.3 --no-unal --no-mixed --no-spliced-alignment for the NAD captureSeq). UMI barcode identification and the deduplication of the mapped data were performed using UMI tools v1.0.1 (69). All work with SAM/BAM files was done using Samtools (v1.9) (70). Gene expression was evaluated using StringTie (71) with the NCBI gene annotation. Differentially expressed genes were identified by the DESeq2 package (72) using a *P* value of 0.05 (adjusted with the Benjamini and Hochberg method) and a fold change of 2. Gene annotations (GO and KEGG ids) were manually transferred from *E. coli* K-12 substr. MG1655 (NCBI:NC_000913) annotation using an in-house script and manual curation. GO and KEGG enrichment analyses were performed using clusterProfiler (73). Statistical analysis was performed using R 4.0.3 (74).

### Northern hybridization

Bacterial cultures were grown in LB medium at 37°C or 24°C till the late exponential growth phase. Total RNA was isolated using RNazol RT (RN 190) (Molecular Research Center, Inc.) as described previously and fractionated on an 8% denaturing polyacrylamide gel with or without acryloylaminophenyl boronic (APB) acid (0.5% final concentration) (75). 2.5× TBE was used for standard denaturing gels and 1 TAE for those with APB. Gels were electroblotted for 2 h at 150 mA on Hybond nitrocellulose membrane (Amersham). After UV cross-linking, membranes were prehybridized in 0.5 M Na_2_HPO_4_ × 2H_2_O, 1 mM EDTA, 7% SDS, and 1% BSA for 2 h at 42°C. Hybridization was performed by adding T4 PNK (Thermo Fisher Scientific) ^32^P-labeled specific oligonucleotides and incubating overnight at 42°C. After hybridization, membranes were washed for 15 min with four different wash solutions containing 0.1% SDS and 2×, 1× (twice), or 0.1 × SSC, respectively. Membranes were exposed to phosphoimager screens that were later visualized using a Fujifilm FLA-5100 scanner and analyzed with Multi Gauge V3.0 software. To determine the relative amount of each sRNA, the membranes were stripped in a boiling 0.1% SDS solution and, starting with the prehybridization step, repeatedly hybridized with ^32^P-labeled oligonucleotides complementary to 5S rRNA. The significance of the obtained results was calculated from three biological replicates using a logistic regression model with PAST3 software after normalization.

### Bacterial motility assays

The bacterial cultures were incubated on a solid LB medium containing an appropriate antibiotic and inducer (arabinose or IPTG, as necessary). The individual colonies were pick-inoculated into semi-solid motility media (1% tryptone, 0.25% NaCl, 0.25% agar). The plates with bacteria were then packed into the airtight, humid containers and incubated at 37°C or 24°C for 16 and 20 h, respectively. The motility was quantified by measuring the diameter of three biological replicates in a halo around the inoculation site.

## Data Availability

The proteomic data are available via the MassIVE database with the identifier MSV000089861. The RNA-seq and NAD captureSeq data have been uploaded to the GEO database (accession number GSE224608).

## SUPPLEMENTAL MATERIAL

Data S1 msystems.00718-23-s0001.xlsx Results of UPLC-MSE analysis of NudC interactome.

Data S2 msystems.00718-23-s0002.xlsx List of NAD-RNAs identified in wt, Δ*nudC*, and Δ*csdA* strains using NAD-Seq.

Data S3 msystems.00718-23-s0003.xlsx Change in gene expression in Δ*nudC* strain compared to wt.

Supplementary material msystems.00718-23-s0004.pdf Interplay between bacterial 5’-NAD-RNA decapping hydrolase NudC and DEAD-box RNA helicase CsdA in stress-responses.

ASM does not own the copyrights to Supplemental Material that may be linked to, or accessed through, an article. The authors have granted ASM a non-exclusive, world-wide license to publish the Supplemental Material files. Please contact the corresponding author directly for reuse.
