# Supplementary material for: Interplay between bacterial 5′-NAD-RNA decapping hydrolase NudC and DEAD-box RNA helicase CsdA in stress responses
Source: mSystems. 2023 Sep 14;8(5):e00718-23. doi: 10.1128/msystems.00718-23 (PMC10654059; doi:10.1128/msystems.00718-23)
Supplement: Supplementary material — Interplay between bacterial 5’-NAD-RNA decapping hydrolase NudC and DEAD-box RNA helicase CsdA in stress-responses. [file msystems.00718-23-s0004.pdf]

SUPPLEMENTARY DATA

Interplay between bacterial RNA 5'-termini deNADding protein NudC and DEAD-box RNA helicase CsdA in stress-responses

Milda Mickutė[1], Renatas Krasauskas[1], Kotryna Kvederavičiūtė[1], Gytė Tupikaitė[1], Aleksandr Osipenko[1], Algirdas Kaupinis[2], Monika Jazdauskaitė[1], Raminta Mineikaitė[1], Mindaugas Valius[2], Viktoras Masevičius[1,3] and Giedrius Vilkaitis[1,*]

[1] Institute of Biotechnology, Life Sciences Center, Vilnius University, Vilnius, LT-10257, Lithuania
[2] Institute of Biochemistry, Life Sciences Center, Vilnius University, Vilnius, LT-10257, Lithuania
[3] Institute of Chemistry, Faculty of Chemistry and Geosciences, Vilnius University, Vilnius, LT-03225, Lithuania

* To whom correspondence should be addressed. Tel: +370 5 2234372; Email: giedrius.vilkaitis@bti.vu.lt

This PDF file includes:

Supplementary Methods
Supplementary Figures S1 to S19
Tables S1 to S6
References (2)

Other Supplementary Materials for this manuscript include the following:

Data S1 to S3

SUPPLEMENTARY METHODS

Quantification of cellular NAD^+

The concentration of cellular NAD^+ was evaluated using NAD/NADH Quantification Kit (Sigma-Aldrich) starting with 5,6×108 CFUs of *E. coli* grown in LB medium at 37°C or 24°C and collected at selected growth points. Samples were lysed as indicated in the protocol and deproteinized by filtering through a Microcon-3 kDa filter (Millipore). The assay was prepared based on manufacturer's recommendations and the concentration of NAD^+ was calculated from three biological and two technical replicates. The significance of the results obtained was evaluated using the Welch t-test and pairwise comparisons.

Construction of plasmids for affinity assays

The concentration of cellular NAD^+ was evaluated using NAD/NADH Quantification Kit (Sigma-Aldrich) starting with $5,6\times10^8$ CFUs of *E. coli* grown in LB medium at 37°C or 24°C and collected at selected growth points. Samples were lysed as indicated in the protocol and deproteinized by filtering through a Microcon-3 kDa filter (Millipore). The assay was prepared based on manufacturer's recommendations and the concentration of NAD^+ was calculated from three biological and two technical replicates. The significance of the results obtained was evaluated using the Welch t-test and pairwise comparisons.

To construct the plasmid pET-28a-NudC-His, NudC encoding gene was firstly amplified from BW25113 genomic DNA using primer pair NudC-Fw/NudC-Rv, cut with NcoI and HindIII and ligated into the pET-28a plasmid pre-cut with the same restriction endonucleases (REs).

A fragment encoding *nudC* gene was cut from pET-28a-NudC-His plasmid using NcoI and HindIII, blunted with T4 polymerase and used to construct the following plasmids: (i) to construct the plasmid pUT18C-NudC it was blunt-ligated into the pUT18C plasmid that was previously cut with HincII and Ecl136II; (ii) to construct the plasmids pUT18-NudC and pKNT25-NudC the fragment was blunt-ligated into the pUT18C or pKNT25 plasmid that was pre-linearised with Ecl136ll; (iii) to construct the plasmid pKT25-NudC the fragment was blunt-ligated into the pKT25 plasmid that was previously cut with PstI and KpnI and blunted with T4 polymerase.

pET28a-NudC-N-His - fragment encoding NudC N-terminal region was amplified from the pET-28a-NudC-His plasmid using primer pair NudC-Fw/NudC-Rv-HindIII. The fragment was cut with NcoI and HindIII and ligated into the plasmid pET-28a-NudC-His that was previously cut with the same REs.

pET28a-NudC-C-His – a fragment encoding NudC C-terminal region was amplified from the pET28a-NudC-N-His plasmid using primer pair NudC-Fw-NcoI/NudC-Rv. PCR product was cut with NcoI and HindIII and ligated into the plasmid pET-28a-NudC-His that was previously cut with the same REs.

pET-15b-His-CsdA - a DNA fragment encoding *csdA* gene was amplified from *E. coli* K-12 MG1655 strain using primer pair CsdA-Fw/CsdA-Rv. PCR product was cut with BamHI and NdeI and ligated into the pET-15b plasmid that was previously cut with the same REs.

pET28a-His-CsdA – a fragment encoding *csdA* gene was cut from the pET-15b-His-CsdA plasmid using NcoI and BamHI. DNA fragment was ligated into the pET-28a plasmid that was previously also cut with the same REs.

pET-28a-His-CsdA-N – a fragment encoding CsdA N-terminal region was amplified from pET28a-His-CsdA plasmid using primer pair CsdA-Fw3/CsdA-444-Pro-Rv. PCR product was blunt-ligated into the pET28a-His-CsdA plasmid that was previously cut with NdeI and BamHI and then blunted with T4 polymerase.

pET28a-His-CsdA-C – a fragment coding CsdA C-terminal region was amplified from pET-28a-His-CsdA plasmid using primer pair NudC-Sacl-Rv2 /CsdA-444-NdeI-Fw. PCR product was ligated into the pET28a-His-CsdA plasmid that was previously cut with NdeI and BamHI.

Construction of BATCH plasmids

pKT25, pKNT25, pUT18C, pUT18 plasmids were restricted using XbaI/SmaI and plasmid backbone is extracted from agarose gel using GeneJET™ Gel Extraction Kit (Thermo Fisher), precipitated with ethanol and dephosphorylated with FastAP (Thermo Fisher) according to manufacturer. Insert is prepared by PCR reaction from E. coli BW25113 strain using Phusion polymerase (Thermo Fisher) with Hfq-XbaI-Fw and Hfq-SmaI-Rv primers, PCR product is cleaved with XbaI and SmaI FD restriction endonucleases (Thermo Fisher) according to manufacturer's recommendations and extracted from agarose gel using GeneJET™ Gel Extraction Kit. 50 ng of insert is ligated with 100 ng of backbone (5 : 1) using T4 DNA ligase (Thermo Fisher) for 2 hours in room temperature. Ligate is transformed in *E. coli* ER1727 competent cells, bacteria were grown on LB medium for 16 hours in 37°C. Colony PCR was performed to select bacteria with inserts with Dream Taq Polymerase (Thermo Fisher) according to manufacturer using pUC19-dir and pUC19-rev primers for pKT25-Hfq or pUC19-rev and pUT18C-Rv primer for pKNT25-Hfq, pUT18-Hfq and pUT18C-Hfq plasmids. Selected colonies were inoculated in LB and grown overnight in 37°C, plasmids were extracted using GeneJet Plasmid Miniprep (Thermo Fisher) and sequenced.

To construct the plasmid pKNT25-NudC-N a fragment encoding NudC N-terminal region was amplified from the pET28a-NudC-N-His plasmid using primer pair NudC-Fw2/NudC-Rv-HindIII. PCR product was cut with HindIII, blunted with T4 polymerase and phosphorylated using T4 Polynucleotide Kinase. The fragment was blunt-ligated into the pKNT25 plasmid that was previously cut with Ecl136ll and Pstl.

pKNT25-NudC-C – a fragment encoding NudC C-terminal region was cut from pET28a-NudC-C-His plasmid with NcoI and HindIII and blunted with T4 polymerase. DNA fragment was blunt-ligated into the pKNT25 plasmid that was previously linearised with Ecl136ll.

pUT18C-CsdA – a fragment encoding *csdA* gene was cut from the pET-15b-His-CsdA plasmid with NdeI and BamHI and blunted with T4 polymerase. The fragment was blunt-ligated into the pUT18C plasmid that was previously cut with HincII and Ecl136II.

pUT18-CsdA and pKNT25-CsdA - fragment encoding *csdA* gene was amplified from the pET-15b-His-CsdA plasmid using primer pair CsdA-HindIII-Fw/CsdA-SacI-Rv. PCR product was cut with HindIII/SacI and ligated into the pUT18 or pKNT25 plasmid pre-cut with the same REs.

pKT25-CsdA - pET-15b-His-CsdA encoding *csdA* gene was cut with NdeI, blunted with T4 polymerase and then cut again with BamHI. Prepared fragment was ligated to pKT25 plasmid, which was pre-linearised with PstI, blunted with T4 polymerase and then cut again with BamHI.

pUT18C-CsdA-N – a fragment encoding CsdA N-terminal region was cut from pET28a-His-CsdA-N plasmid with NdeI and BamHI and blunted with T4 polymerase. DNA fragment was blunt-ligated into the pUT18C plasmid that was previously linearised with Ecl136ll.

pUT18C-CsdA-C - a fragment encoding CsdA C-terminal region was cut from pET28a-His-CsdA-C plasmid with NdeI and BamHI and blunted with T4 polymerase. DNA fragment was blunt-ligated into the pUT18C plasmid that was previously linearised with Ecl136ll.

pACYC184-NudC-His - a fragment encoding *nudC* gene with six histidine residues was amplified from pET28a-NudC-His plasmid using primer pair NudC-Fw2/NudC-SacI-Rv2, cut with KpnI, and blunted with T4 polymerase. This fragment was blunt-ligated to pACYC184 plasmid that was previously amplified with a primer pair pACYC184-SacI-Fw/pACYC184-Rv2, cut with SacI and blunted with T4 polymerase.

Plasmids and primers used in this work are provided in Supplementary Table S4 and Supplementary Table S5, respectively.

NudC activity assay

The hydrolase activity of NudC was measured in a reaction buffer containing 25 mM Tris/HCl (pH 7.5), 50 mM NaCl, 50 mM KCl, 10 mM MgCl2, 1mM DTT, 0,1 mM BSA, 1U/µl RiboLock (Thermo Fisher Scientific), 0,2 µM in vitro transcribed 5'-PPP- or 5'-NAD-RNA and 2 µM of NudC-His. After 30 min of incubation at 37°C samples were fractionated on a 13% denaturing PAA gel and visualized after staining with SybrGold (Thermo Fisher Scientific).

Construction of double *E. coli* BW25113 Δ*nudC*Δ*csd*A mutant strain

Double *E. coli* BW25113 Δ*nudC*Δ*csd*A mutant strain was constructed by eliminating the kanamycin resistance cassette from *E. coli* BW25113 Δ*nudC* (BW25113 Δ*nudC*::FRT-Km^R-FRT) and replacing its *csdA* gene with kanamycin cassette from *E. coli* BW25113 Δ*csdA*. The construction was performed using Quick & Easy *E. coli* Gene Deletion Kit (Gene Bridges GmbH). After chemically transforming *E. coli* BW25113 Δ*nudC* with 708-FLPe plasmid, the removal of kanamycin resistance cassette was confirmed by PCR using NudC-Km-Fw and NudC-Km-Rv primers. The fragment encoding the kanamycin resistance cassette at the *csdA* gene site was PCR amplified from *E. coli* BW25113 Δ*csdA* (BW25113 Δ*csdA*::Km^R) strain with CsdA-Fw/CsdA-Rv primer pair.

Plasmid construction for analysis of bacteria growth phenotype

For the pACYC184-NudC plasmid, the NudC-encoding gene was PCR-synthesized using BW25113 genomic DNA and NudC_prom_Fw/NudC_prom_Rv primers pair. PCR product was ligated into the pACYC184 plasmid that was previously cut with EheI and Bus15I.
To prepare the plasmid pACYC184-DeaD a fragment coding CsdA region was amplified from pET28a-His-deaD plasmid using primers pair CsdA-Fw4/NudC-SacI-Rv2 and cut with BamHI. PCR product was blunt-ligated into the pACYC184 plasmid that was PCR-amplified with pACYC184-SacI-Fw/pACYC184-Rv2 primer pair and cut with SacI.

Analysis of bacteria growth phenotype on LB agar medium

The growth of *E. coli* strains under different conditions was examined by a spotting assay. Overnight cultures were serially diluted from 1 to 10^{-5} final absorbance at 600 nm. 3 µL of each dilution were spotted onto standard LB agar medium plates. The experiments were also performed on LB medium containing 2mM H_2O_2 or different pH level (pH=5; pH=9) and repeated for three times. The formation of colonies on the agar was observed after 12 h, 18 h and 16 h incubation at 37°C, 24°C or 42°C temperature, respectively.

Plasmid construction for protein localization analysis

Plasmid pZA_ecfp was made by amplifying the *ecfp* gene from pECFP and blunt cloning it into pZA-3 plasmid. Plasmids pZA-NudC_ecfp, pZA_CsdA_ecfp, pL9_ecfp, were blunt-cloned by amplifying the respective gene as well as *ecfp* gene, overlapping and ligating them into pZA-3 plasmid via KpnI site. To construct the plasmid pZS_RNaseE_eyfp_tetR, RNaseE encoding gene was firstly amplified, overlapped with *eyfp,* cut with Bsp120I, and blunt-ligated into pZS24-MCS1 plasmid via KpnI site. The plasmid pZS_eyfp_tetR was obtained by blunt-cloning ApaI cut *eyfp* into pZS24-MCS1 plasmid via KpnI. Both pZS plasmids were then used as a recipient for the tetracycline gene that was cut with XbaI and Eco88 from pACYC184 plasmid and blunt ligated via NdeI site. Plasmid pZA-NudC was obtained by cloning amplified *nudC* gene into pZA-3 plasmid via KpnI site. Plasmids and primers used in the work are provided in Supplementary Table S4 and Supplementary Table S5, respectively.

Analysis of bacterial growth curves

Overnight cultures of *E. coli* grown in 5 mL LB media at 37 °C were diluted with fresh media with the selective antibiotic and an inducer (arabinose or IPTG, as necessary) to a cell density of 10^7 CFU/mL, transferred to the 96-well polystyrene plates (Nerbe) in a total volume of 200 µl per well, and grown at 37°C or 24°C with shaking at 200 rpm. The growth of cultures was monitored using a Synergy H4 microplate reader. The experiment was repeated three times in three technical replicates.

Transmission electron microscopy (TEM)

Overnight bacterial cultures were diluted 100-fold with fresh liquid motility media containing 10 g/L tryptone and 2.5 g/L NaCl. Then, an appropriate inducing agent (arabinose and/or IPTG) was added. The cultures were grown to mid-log (OD_{600} ~ 0.5), centrifuged at 1,000 × g for 10 min, and gently resuspended in 50 µl PBS. Suspensions were absorbed onto formvar carbon-coated copper grids for 5 min. Excess liquid was removed using filter paper before staining with 2% uranyl acetate (pH 4.5). Samples were air-dried and examined in a Tecnai G2 F20 X-TWIN (FEI) transmission electron microscope equipped with an 11 MPix ORIUS SC1000B (Gatan) CCD camera. At least 3 images representing the population seen were taken.

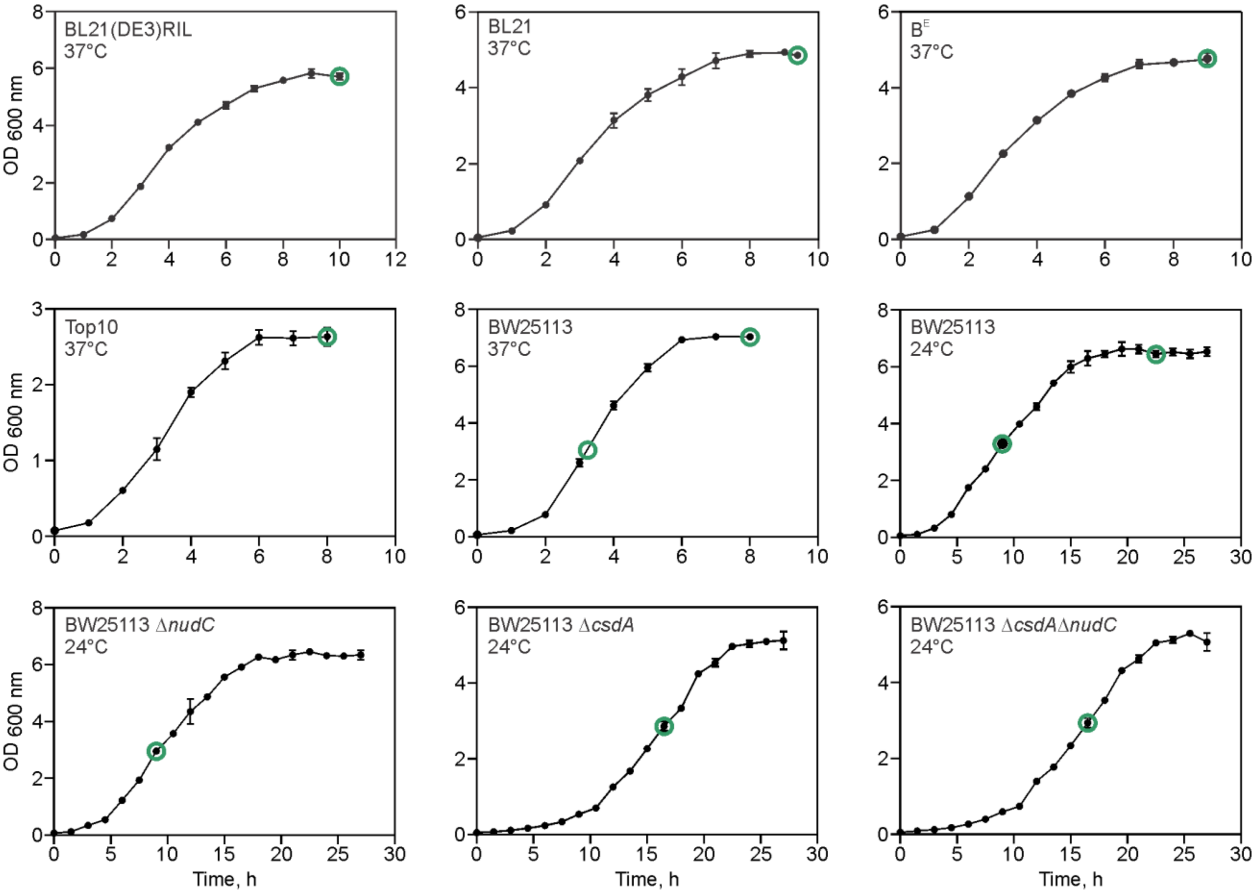

Supplementary Figure S1. The growth curves of *E. coli* strains used for the quantification of 5'-NAD$^+$-RNA and cellular level of NAD$^+$. Mean values ± SD were obtained from three biological replicates. Green circles indicate biomass collection points.

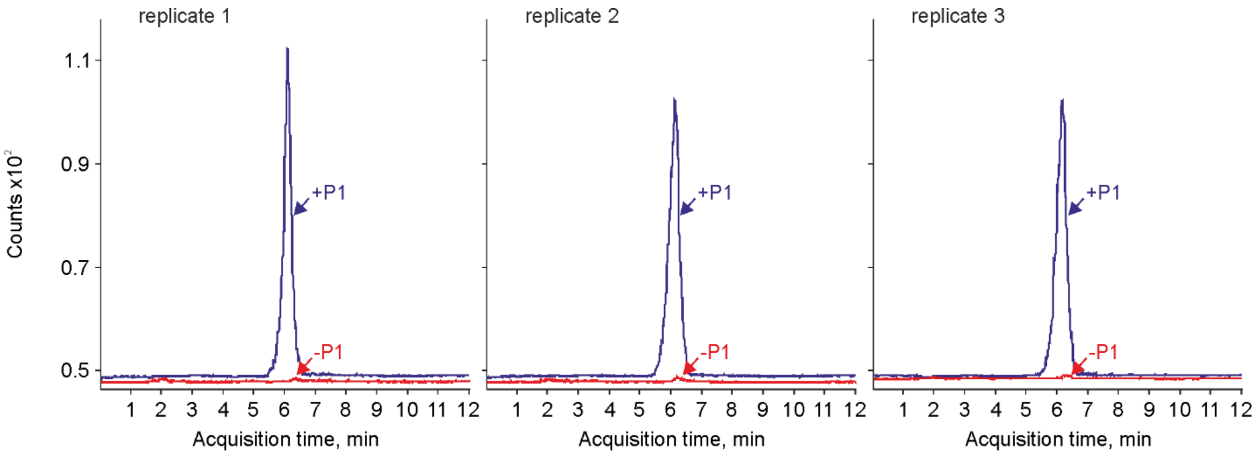

Supplementary Figure S2. Exemplary HPLC-MS/MS measurements, which were used for the quantification of 5'-NAD$^+$-RNA in *E. coli* BL21(DE3)RIL presented in Figure 1. The intensity of the modification-specific ion transition (m/z 664.1 → 542.1) was recorded. The results for three biological replicates, both treated (+P1) and untreated (-P1) with Nuclease P1, are presented.

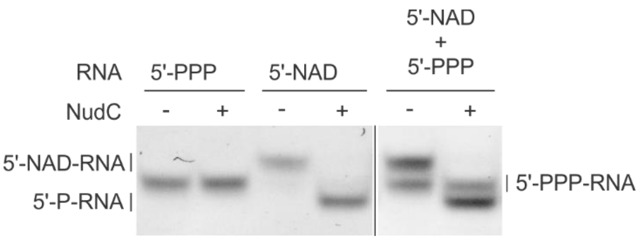

Supplementary Figure S3. The hydrolase activity of purified NudC-His bait-protein used in pull-down experiments. 0,2 µM of *in vitro* transcribed RNA was incubated with 2 µM of NudC for 30 min at 37°C and analysed on 13% PAA gel. At least three independent replicates were made and the representative results are shown.

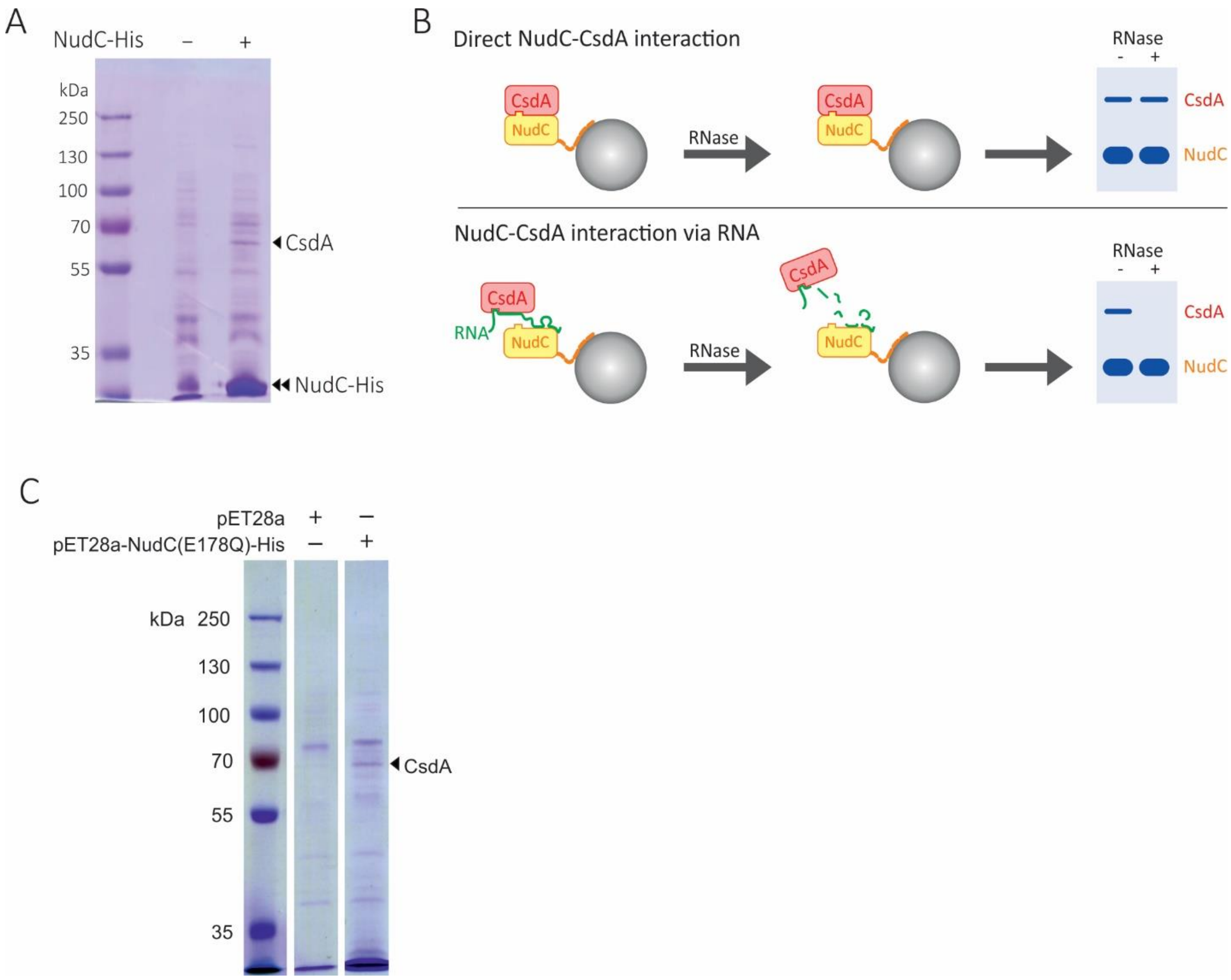

Supplementary Figure S4. Investigation of NudC interaction with CsdA. (A) NudC-His added to lysate pulled-down CsdA protein in *E. coli* BW25113 strain at 16°C temperature. Two independent experiments were conducted and the representative results are shown. **(B)** The scheme of the experiment presented in Figure 2B. Lysate-extracted proteins coupled to sepharose beads are treated with RNase A and analysed on SDS-PAGE. In case of RNA-mediated interaction CsdA protein would not be extracted with the beads. **(C)** NudC(E178Q)-His expressed in *E. coli* BW25113 strain pulled-down CsdA protein at 16°C temperature. The experiment was repeated twice and the representative results are provided.

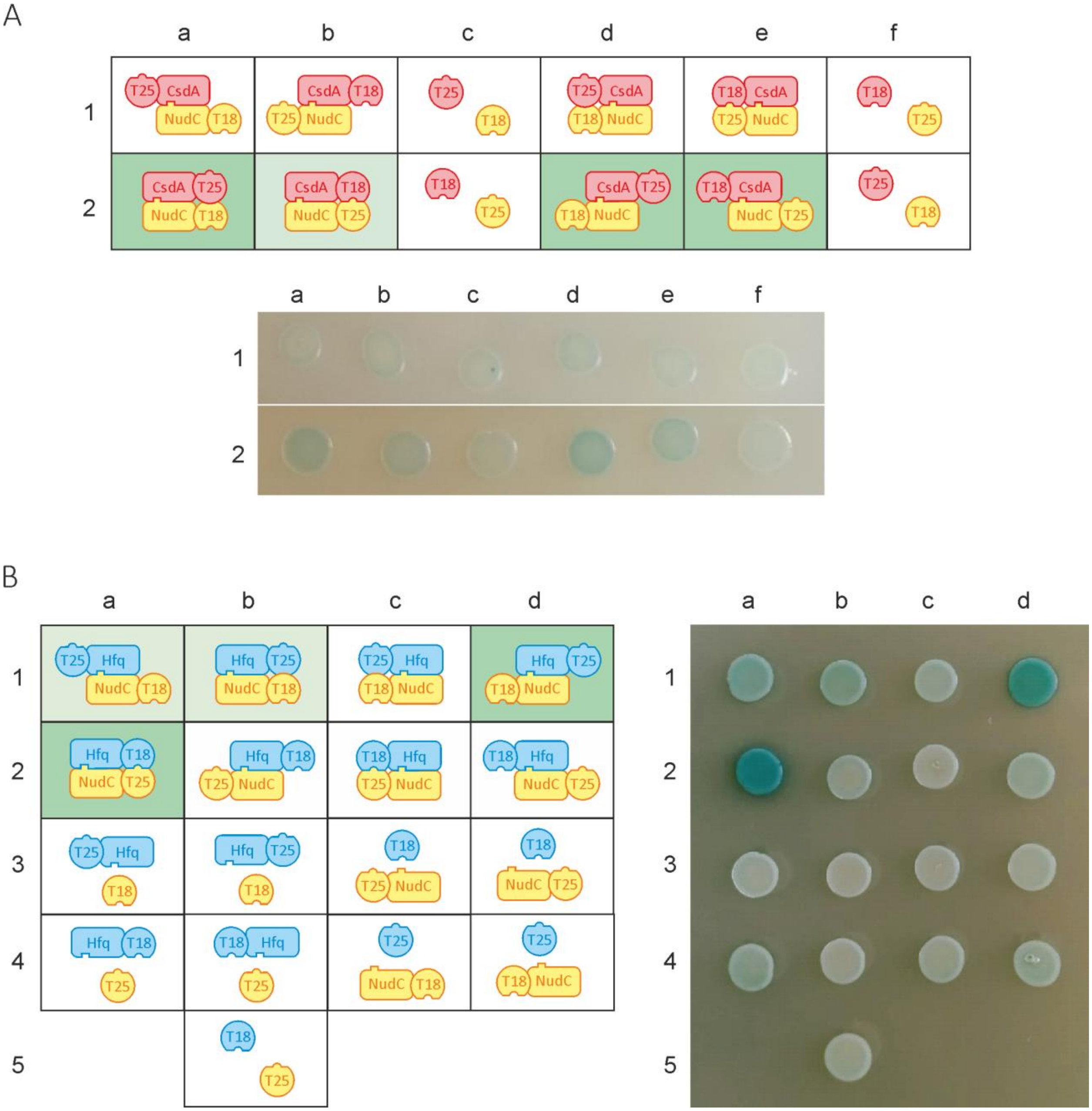

Supplementary Figure S5. BATCH analyses of variant fusion constructs of full-length NudC, CsdA and Hfq. (A) NudC interacts with CsdA. The adenylate cyclase activity using T18 and T25 subunits was reconstituted in samples a2, b2, d2, e2. At least three independent transformations were made and representative results are shown. **(B)** NudC interacts with Hfq (a1, b1, δ1, a2).

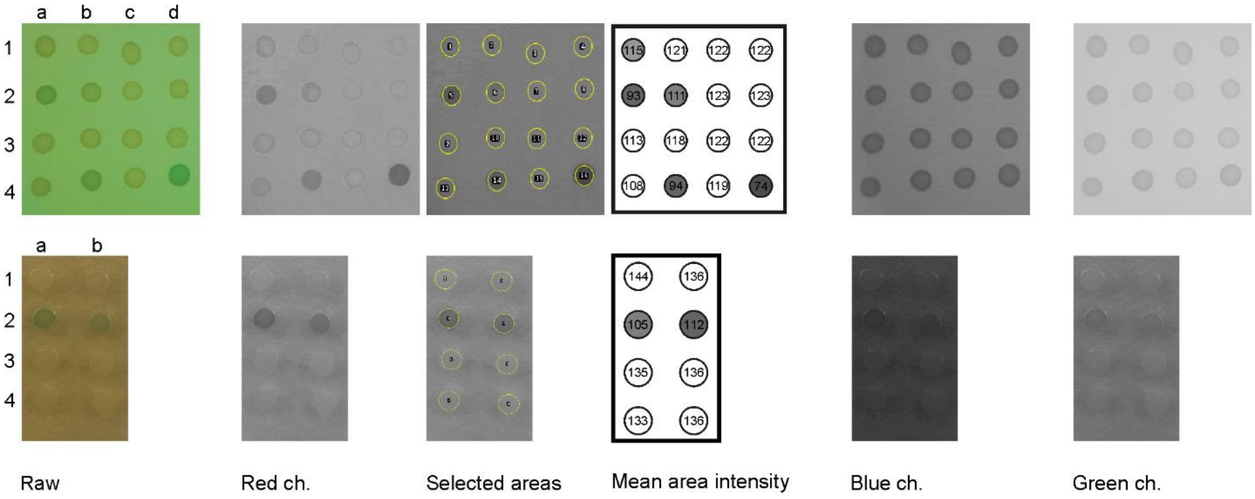

Supplementary Figure S6. Analysis of the BATCH images depicted in Fig. 2C (top row) and Fig. 3A (bottom row) using Fiji software version 1.53c (*1*)**.** The JPG images were split into red, green, and blue image channels. The entire colony in the red channel was selected using an oval tool, and the pixel intensity of its area was evaluated. The figure shows the original image, its colour channels, the selected areas for assessment, and the quantitative values of colonies in the red channel.

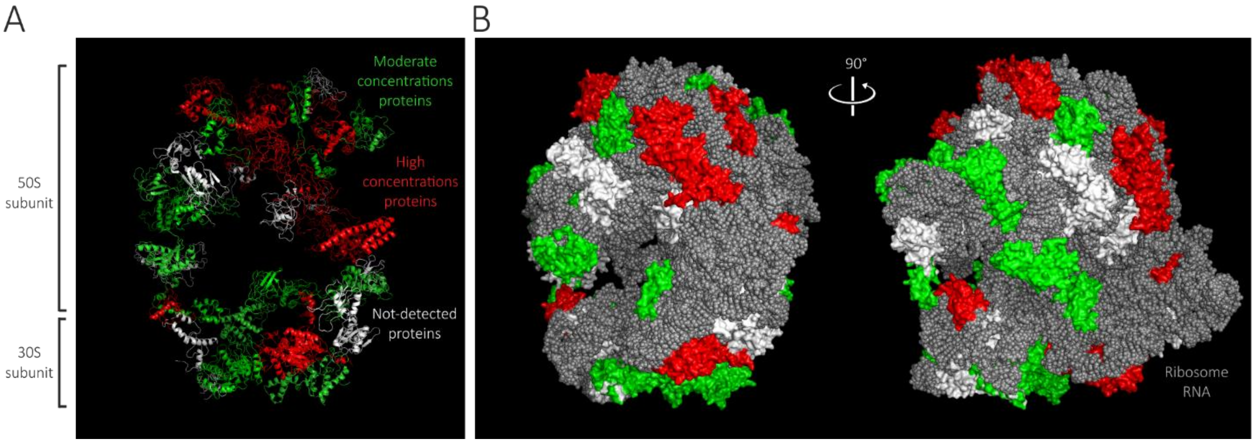

Supplementary Figure S7. Ribosome proteins determined in the NudC interactome. (A) About 80% of ribosome proteins were co-purified with NudC-His expressed in *E. coli* BL21-CodonPlus (DE3)-RIL cells at 16°C. UPLC-MS^E-detected ribosome proteins are depicted in red (>5 fmol) and green (0.5-5 fmol), not-detected – in white. **(B)** The model including ribosomal RNAs in grey. The structure of 70S elongation competent ribosome was taken from RCSB Protein Data Bank (PDB: 6O9J).

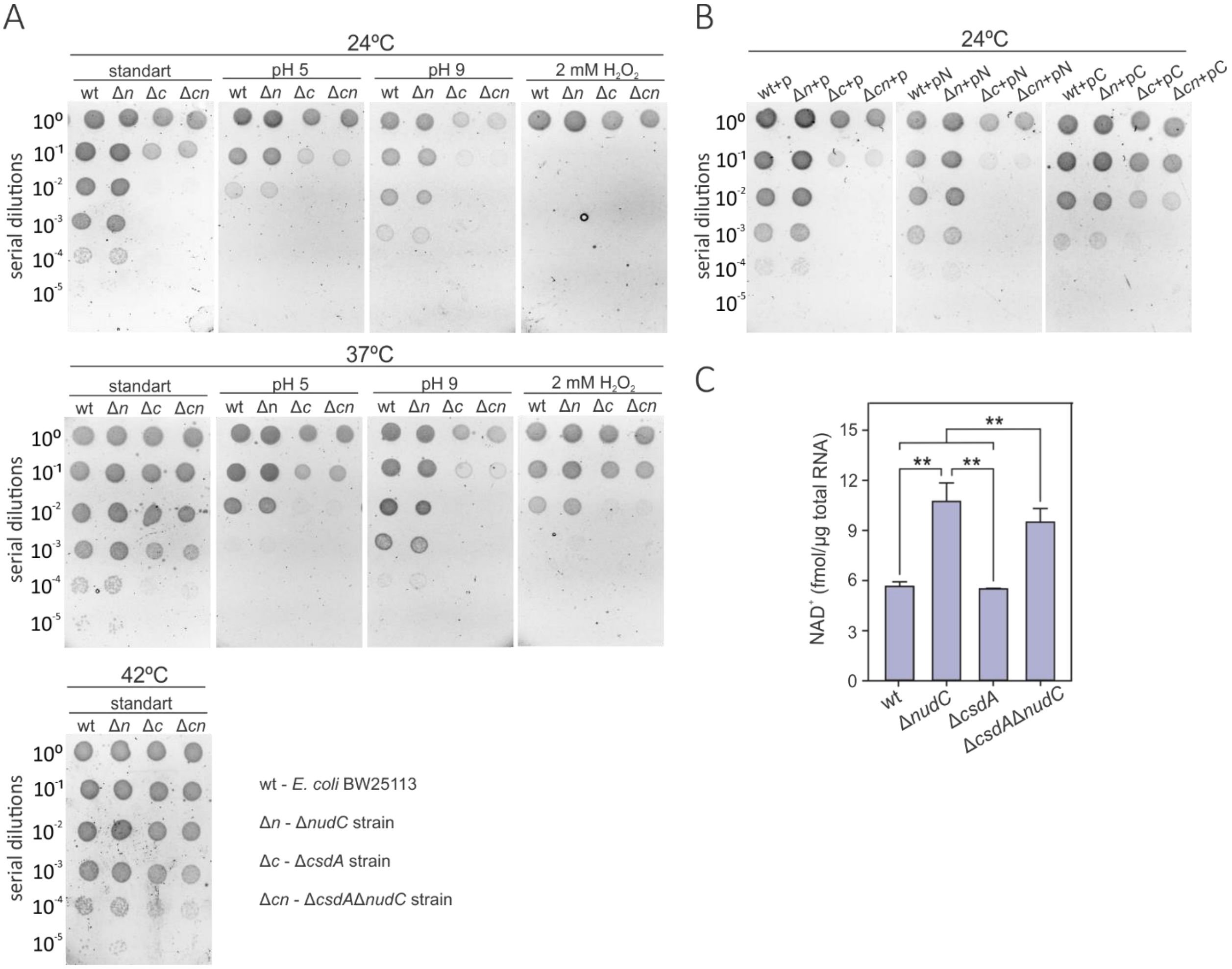

Supplementary Figure S8. Analysis of growth phenotype and the total level of 5'-NAD$^+$-RNA modification in *E. coli* BW25113 and its mutant strains. (A) The effect of temperature, pH and H_2O_2 on the bacteria growth. Serial 10-fold dilutions of over-night cultures were spotted on LB agar medium and incubated at indicated temperatures for 18, 12 and 16 h at 24°C, 37°C and 42°C, respectively. **(B)** The expression of *csdA* restores the reduced growth of Δ*csdA* and Δ*csdA*Δ*nudC* strains. Bacteria transformed with empty pACYC184 vector (p), pACYC184-NudC (pN) and pACYC-CsdA (pC) plasmid were grown on LB agar medium for 20 h at 24°C. **(C)** HPLC-MS analysis of *E. coli* BW25113 strains grown at 24°C up to an exponential growth stage. The significance was calculated using Welch t-test and pairwise comparisons **p<0.01. Strains: wt – *E. coli* BW25113, Δ*n* – Δ*nudC*, Δc – *ΔcsdA*, *Δcn* - Δ*csdA*Δ*nudC*

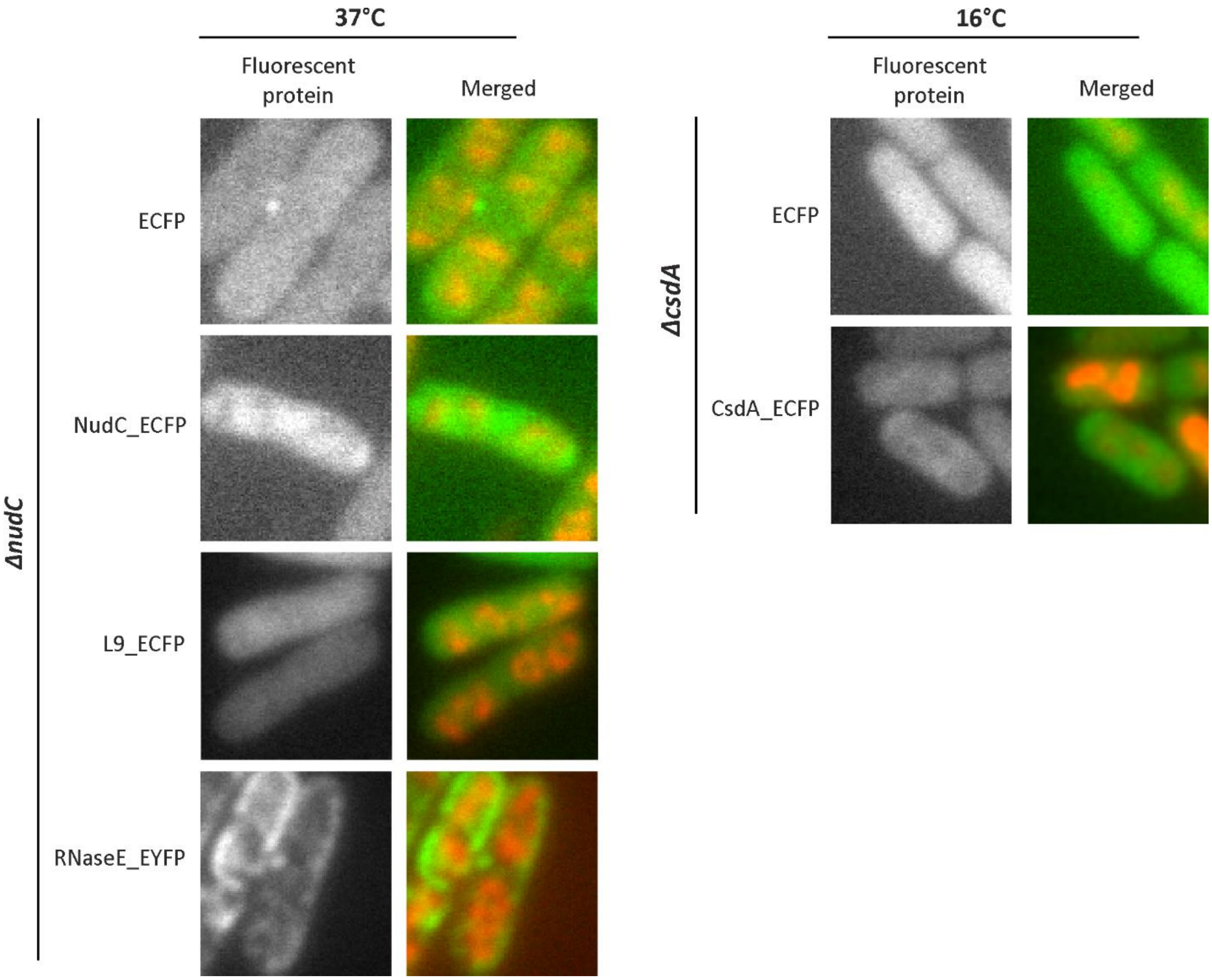

Supplementary Figure S9. Cellular localization of ECFP and EYFP tagged ribosome and degradosome components in wild-type strain BW25113 and its Δ*nudC* and Δ*csdA* mutant derivatives deficient in NudC and CsdA, respectively. The strains were complemented with inducible plasmids encoding either fluorophore (EYFP) or C-terminal fluorophore (ECFP or EYFP) tagged versions of RNaseE and ribosomal L9 proteins. Constructs were induced with varying concentrations of arabinose. Localisation was evaluated in either 37°C **(A)** or after 30 min shock in 16°C **(B)**. Representative pictures out of two independent replicas are shown. Nucleoids were stained with DAPI. Scale bar: 5 µm.

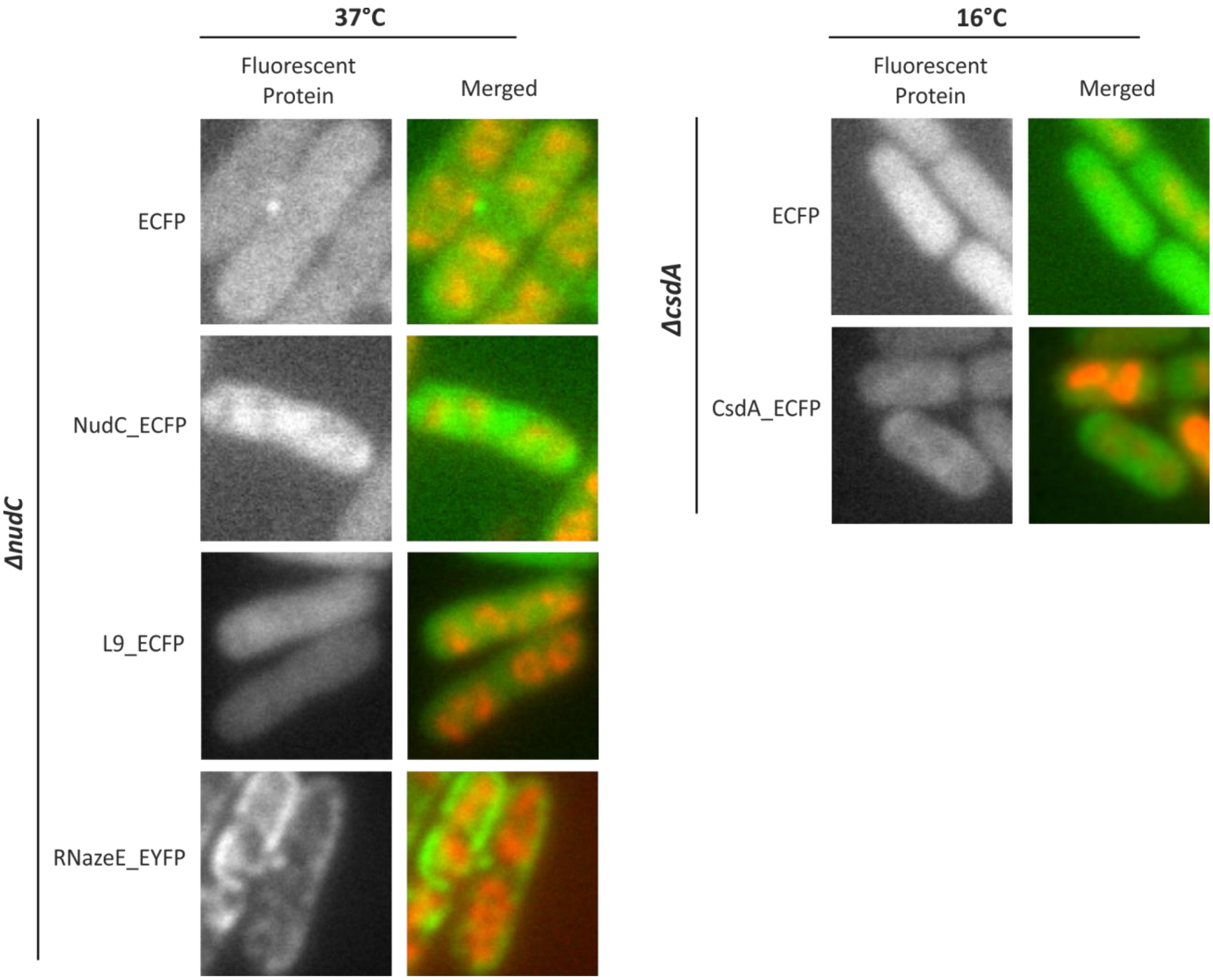

Supplementary Figure S10. Magnification of certain areas within the selected images presented in the Figure 4. Fluorescent protein images display subcellular distribution of fluorescent protein ECFP alone or C-terminal ECFP fused to NudC, CsdA and L9. RNaseE was fused to EYFP. Merged images show both fluorophore and DNA-specific DAPI stained nucleoids.

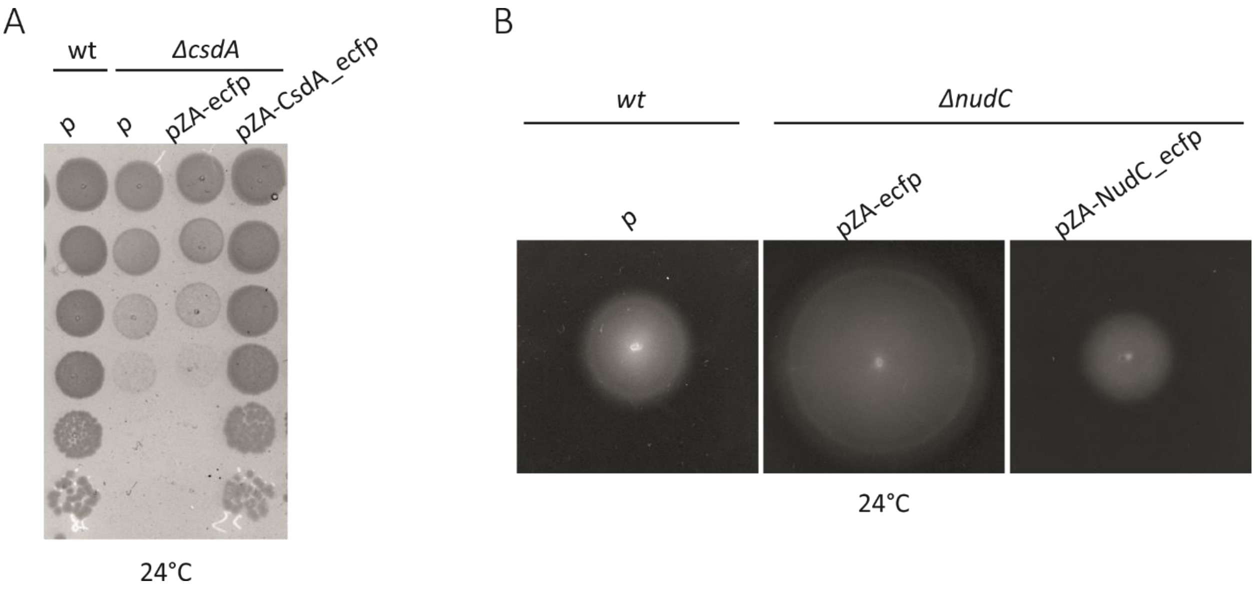

Supplementary Figure S11. Phenotypic rescue of growth and swimming defects by chimeric CsdA-ECFP NudC-ECFP proteins, respectively. (A) Serial dilutions of overnight grown wt and *ΔcsdA* cells were spotted on LB agar plates containing 0.02% arabinose and incubated overnight at 24°C. **(B)** Cells were spotted on soft agar (0.25%) and incubated for 24h at 24°C. NudC-ECFP expression in *ΔnudC* strain was induced by adding 0.02% arabinose. p – pZA-3 vector; pZA-ecfp encodes fluorescence protein only; pZA-NudC_ecfp and pZA-CsdA_ecfp encode NudC and CsdA tagged with ECFP, respectively.

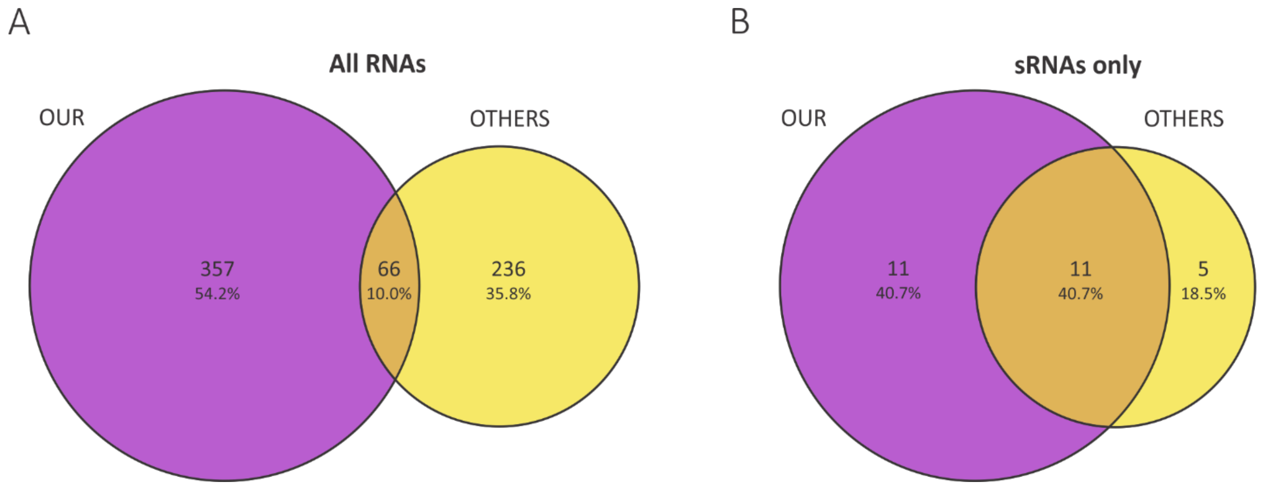

Supplementary Figure S12. Comparison of 5'-NAD-RNAs identified in this study with combination of three earlier published datasets. (A) Venn diagram depicting overlap of all RNAs. **(B)** Overlap of sRNAs.

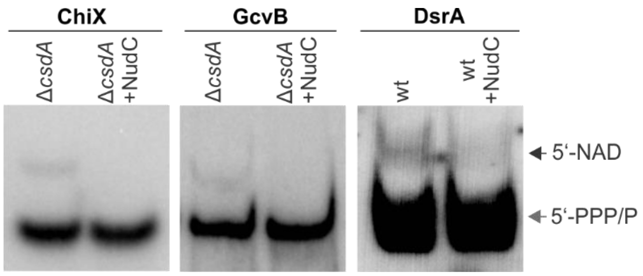

Figure 13. NudC catalyzed decapping confirmed the presence of 5’-NAD modification on ChiX, GsvB and DsrA sRNAs. Intact or treated with NudC hydrolase (+NudC samples) 6 µg of total RNA purified from *E. coli* BW25113 (wt) and its derivative strain Δ*csdA* grown at 24°C was fractionated on an 8% denaturing PAA gel copolymerized with 0.5% acryloylaminophenyl boronic acid (APB). Individual sRNAs were detected by Northern blotting.

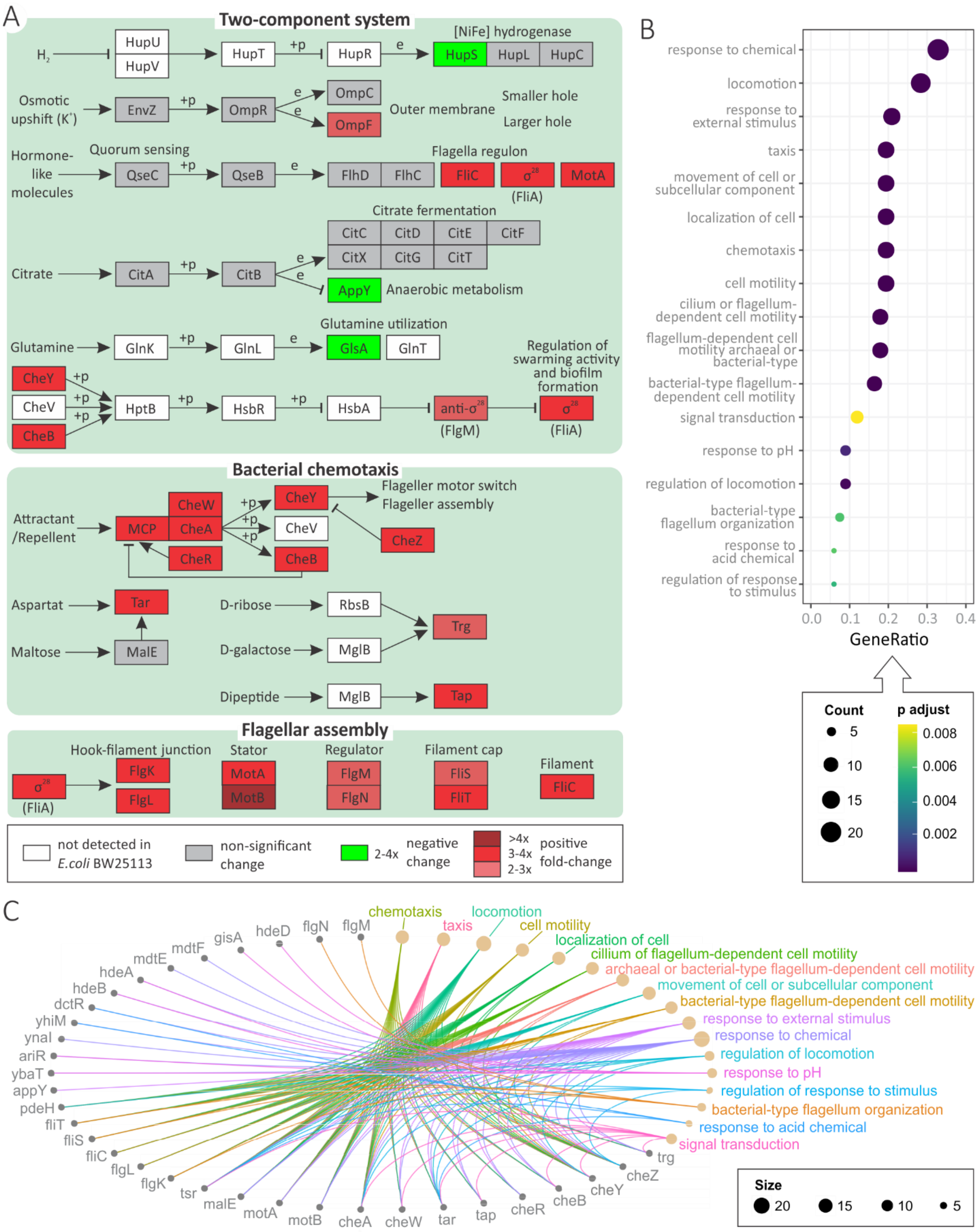

Supplementary Figure S14. Overview of the RNA sequencing results. (A) Affected genes in KEGG gene pathways. **(B)** GO pathway functional classification of all the differentially expressed transcripts. **(C)** GO umbrella analysis of all the detected genes. Adj p-values <0.05 and log2 fold-change cut-off of ≥1.

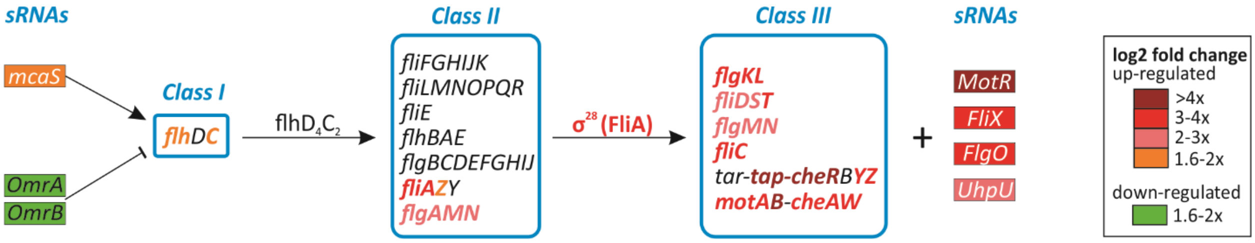

Supplementary Figure S15. Operons of flagellar regulatory cascade affected in Δ*nudC* strain. Differentially regulated protein coding genes are depicted in bold (fold-change level is denoted by the colours), sRNAs – in colour-graded rectangles (significance p_{adj} value cut-off of <0.05). The operons are assigned to three temporally regulated transcriptional classes: I - early, II - middle, and III – late.

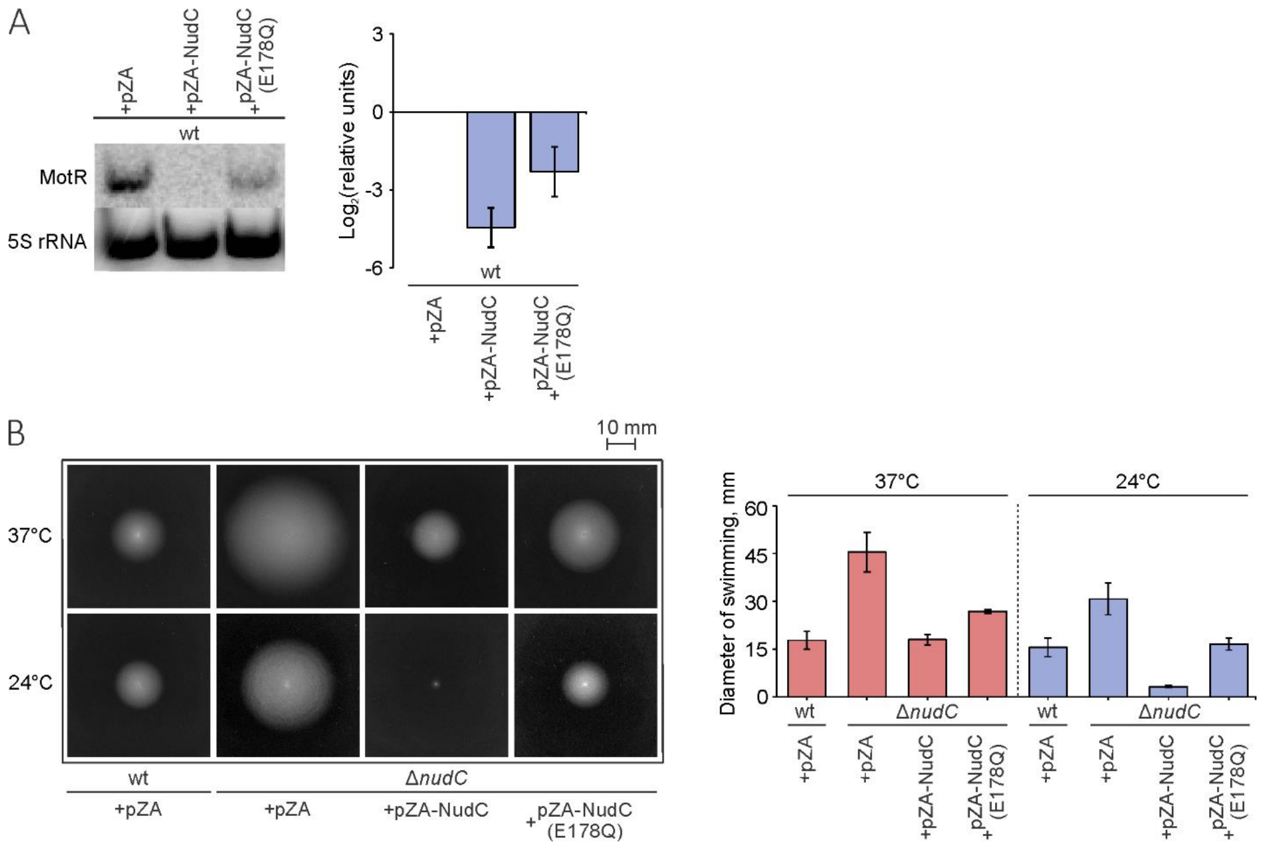

Supplementary Figure S16. Effect of NudC(E178Q) protein on bacterial motility and MotR sRNA expression. **(A)** The amount of MotR sRNA decreases in wild-type BW25113 when NudC and NudC(E178Q) proteins are expressed. 5S rRNA was used as a quantity control. Three independent biological replicates were used to calculate the mean ± SD. **(B)** Images of a colony spreading of strains with empty pZA vector, plasmid-mediated expression of NudC or NudC(E178Q) protein in wild-type BW25113 and Δ*nudC* knockout strain. Cells were spotted on soft agar (0.25%) with IPTG and incubated for 16 h and 20 h at 37°C and 24°C, respectively. The mean swimming diameter ± SD for each strain was calculated from three biological replicates.

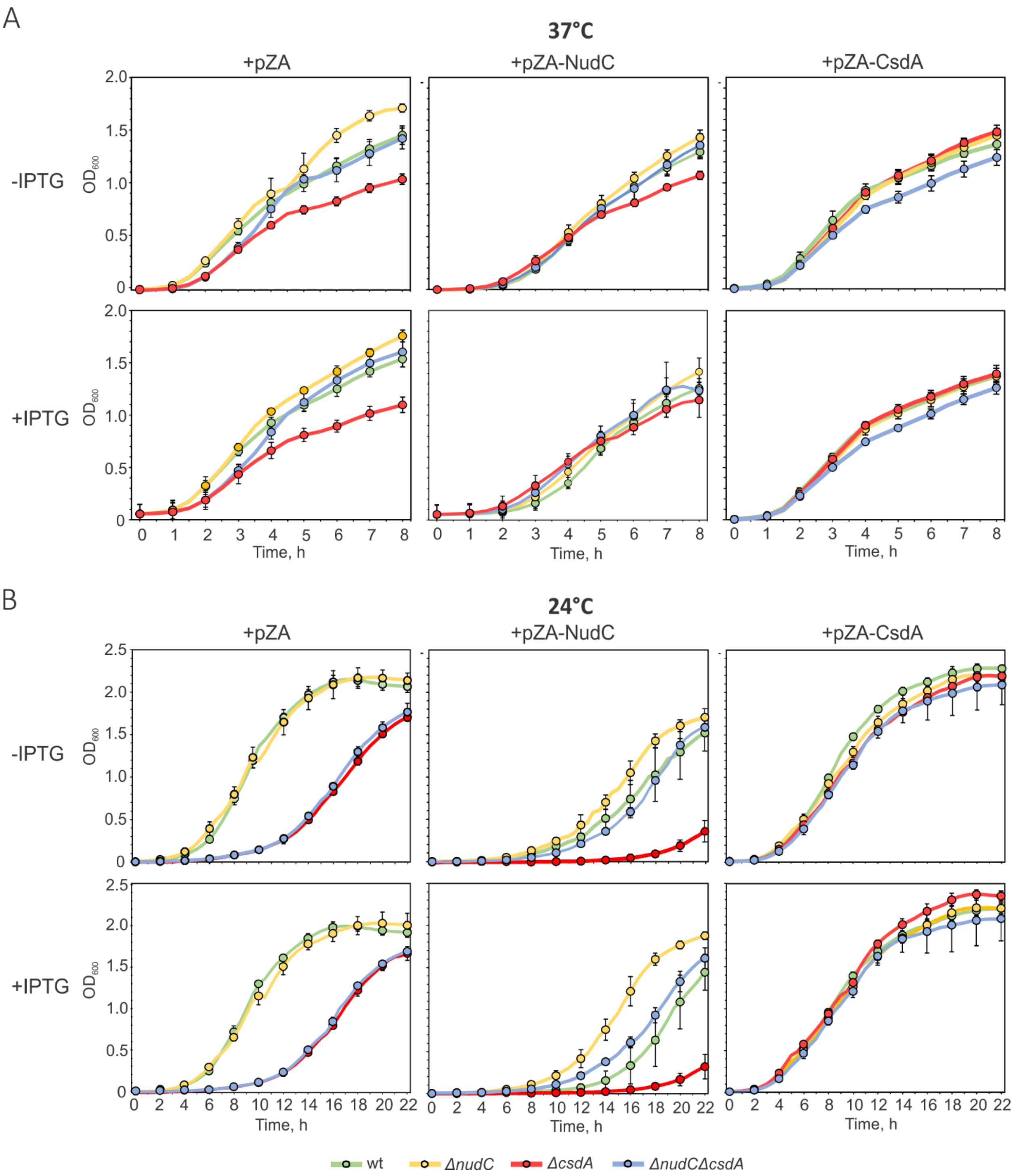

Supplementary Figure S17. Restoration of growth of *E. coli* BW25113 and its mutant strains at 37°C (A) or 24°C (B) temperature. BW25113 wild-type (wt) or its variants with deletions (Δ*csdA*, Δ*nudC*, and Δ*nudC*Δ*csdA*) were transformed with plasmid pZA-3 (left row), pZA-NudC (centre row) or pZA-CsdA_ecfp (right row). The curves were obtained from diluted overnight cultures in LB media without (control) or with inductor IPTG (these conditions correspond to those used in the Figure 8).

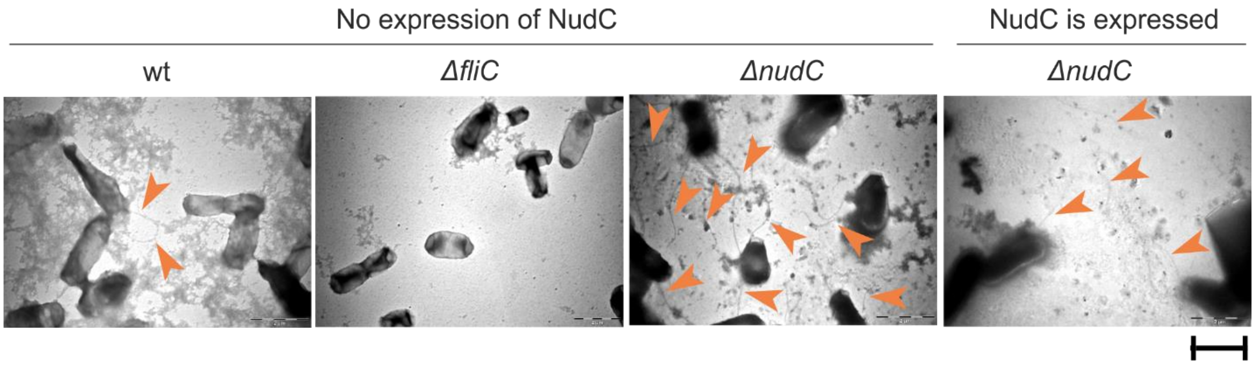

Supplementary Figure S18. Detection of bacterial flagella in wild-type BW25113 and its mutant strains by transmission electron microscopy (TEM). The *ΔnudC* strain was evaluated without or with complementing plasmid pZA-NudC encoding NudC. The *ΔfliC* mutant lacking flagella was used as a negative control. All cells were obtained from the edges of moving colonies on motility plates. Orange arrows mark bacterial flagella. Scale bar: 2 µm.

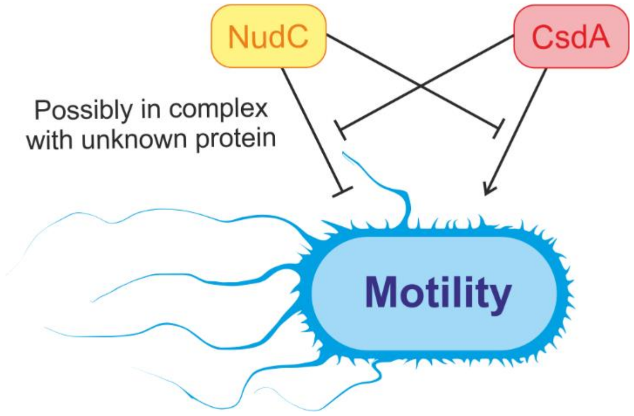

Supplementary Figure S19. The proposed model of the NudC and CsdA roles in regulation of bacterial motility. Free or in complex with unknown protein NudC is postulated to trigger decrease in bacterial motility (it is possible that dose effect occurs). In contrast, CsdA promotes *E. coli* motility. The effect of both proteins is neutralized when NudC forms a complex with CsdA.

Supplementary Table S1. 5'-NAD+-RNA and cellular levels of NAD+ detected using HPLC-MS analysis and colorimetric quantification, respectively. The results are the mean of at least three biological replicates.

**Strain**	**5'-NAD$^+$-RNA ± SD (fmol/µg total RNA)**			
	Stationary growth phase		Exponential growth phase	
	37°C	24°C	37°C	24°C
**BL21(DE3)RIL**	26.6 ± 1.3			
**BL21**	14.5 ± 2.9			
**B^E**	9.7 ± 2.2			
**Top10**	4.2 ± 1.1			
**BW25113 (wild type and mutant derivatives):**				
**wt**	2.9 ± 0.2	8.2 ± 1.3	3.9 ± 0.4	5.0 ± 0.3
**Δ*nudC***				10.7 ± 1.2
**Δ*csdA***				5.5 ± 0.1
**Δ*nudC*Δ*csdA***				9.5 ± 0.8
	**Free cellular NAD$^+$ ± SD (pmol/10^8 cells)**			
	Stationary growth phase		Exponential growth phase	
	37°C	24°C	37°C	24°C
**BW25113**	100.4 ± 10.1	93.0 ± 8.9	43.5 ± 10.6	100.6 ± 11.4

Supplementary Table S2. Changes in 5'-NAD$^+$ modification level and sRNA amount under different temperature and genetical background. The means were obtained from three biological replicates analysed by Northern blotting. Fold changes were calculated relative to wild type BW25113 cells at 24°C (◌).

		ChiX			DsrA			GcvB		
		5'-NAD-sRNA		sRNA	5'-NAD-sRNA		sRNA	5'-NAD-sRNA		sRNA
	**Strain**	Mean, % [1]	Fold ± SD	Fold ± SD	Mean, % [1]	Fold ± SD	Fold ± SD	Mean, % [1]	Fold ± SD	Fold ± SD
37°C	**wt**	4.7	-1.1 ± 0.1	1.6 ± 0	4.6	-2.0 ± 0.2	-6.5 ± 1.3	5.0	1.1 ± 0.1	-5.6 ± 2.4
24°C	**wt**	5.0	◌	◌	9.1	◌	◌	4.7	◌	◌
	ΔnudC	2.1	-2.7 ± 1.5	1.6 ± 0.3	14.5	1.6 ± 0.2	-1.5 ± 0.4	8.8	1.8 ± 0.6	-3 ± 1.2
	ΔcsdA	3.0	-1.8 ± 0.5	-1.6 ± 0.4	1.8	-5.3 ± 1.1	-1.8 ± 0.4	5.4	1.0 ± 0.7	3.7 ± 1
	ΔnudCΔcsdA	1.9	-2.8 ± 0.8	-1.1 ± 0.2	12.5	1.4 ± 0.2	-2.0 ± 0.3	3.8	-1.5 ± 0.6	-2.0 ± 2.1

[1] $p \leq 0.001 - 0.05$

Supplementary Table S3. Effect of *nudC* and *csdA* on bacterial motility at 37°C and 24°C. The motility diameter (mean ± SD, mm) was measured as shown in Figure 8.

	37°C			24°C		
**Strain**	**+pZA**	**+pZA-NudC**	**+pZA-CsdA**	**+pZA**	**+pZA-NudC**	**+pZA-CsdA**
**wt**	17.8 ± 2.8	5.2 ± 0.4	11.5 ± 1.3	15.5 ± 2.9	1.6 ± 0.1	25.9 ± 4.5
ΔnudC	45.4 ± 6.2	18.0 ± 1.6	40.9 ± 2.8	30.7 ± 5.0	3.2 ± 0.3	34.2 ± 4.4
ΔcsdA	26.5 ± 1.7	18.0 ± 4.3	36.6 ± 4.1	7.2 ± 1.0	0.8 ± 0.1	33.0 ± 5.8
ΔnudCΔcsdA	25.5 ± 0.8	14.7 ± 4.6	39.5 ± 3.3	7.2 ± 0.3	1.0 ± 0.2	35.0 ± 4.9

Supplementary Table S4. List of plasmids used in the current work.

Name	Source	Purpose
708-FLPe	Gift from Prof. Dr. Rolandas Meškys	Δ*nudC*Δ*csdA* mutant strain construction
pACYC184	Laboratory collection	Source of tetracycline resistance gene
pACYC184-CsdA	This work	Complementation
pACYC184-NudC	This work	Complementation
pACYC184-NudC-His	This work	Affinity-tag pull-down
pCasper-4xMTo-EYFPb	Gift from Dr. Danguolė Žiogienė	Source of *eyfp*
pECFP	Gift from Dr. Danguolė Žiogienė	Source of *ecfp*
pET-15b	Laboratory collection	BACTH assay
pET-15b-His-CsdA	Laboratory collection	Source of CsdA protein coding gene
pET28a	Laboratory collection	PUMC
pET28a-His-CsdA	Laboratory collection	Source of CsdA protein coding gene
pET28a-His-CsdA-C	This work	Source of csdA C-terminal region
pET28a-His-CsdA-N	This work	Source of csdA N-terminal region
pET28a-NudC-C-His	This work	Source of nudC C-terminal region
pET28a-NudC-His	Laboratory collection	Affinity-tag pull-down and BACTH assay
pET28a-NudC-N-His	This work	Source of nudC N-terminal region
pKNT25	Gift from Dr. Julija Armalytė	BACTH assay
pKNT25-CsdA	This work	BATCH assay
pKNT25-CsdA-C	This work	BATCH assay
pKNT25-CsdA-N	This work	BATCH assay
pKNT25-Hfq	This work	BATCH assay
pKNT25-NudC	This work	BATCH assay
pKNT25-NudC-C	This work	BATCH assay
pKNT25-NudC-N	This work	BATCH assay
pKT25	Gift from Dr. Julija Armalytė	BACTH assay
pKT25-CsdA	This work	BATCH assay
pKT25-Hfq	This work	BACTH assay
pKT25-NudC	This work	BATCH assay
pRedET	Gift from Prof. Dr. Rolandas Meškys	Δ*nudC*Δ*csdA* strain construction
pUT18	Gift from Dr. Julija Armalytė	BACTH assay
pUT18C	Gift from Dr. Julija Armalytė	BACTH assay
pUT18C-CsdA	This work	BATCH assay
pUT18C-CsdA-C	This work	BATCH assay
pUT18C-CsdA-N	This work	BATCH assay
pUT18C-Hfq	This work	BACTH assay
pUT18C-NudC	This work	BATCH assay
pUT18-CsdA	This work	BACTH assay
pUT18-Hfq	This work	BACTH assay
pUT18-NudC	This work	BATCH assay
pZA-3	Laboratory collection	(*2*)
pZA-CsdA_ecfp	This work	Localisation and motility
pZA-ecfp	This work	Localisation

pZA-L9_ecfp	This work	Localisation
pZA-NudC	This work	Complementation
pZA-NudC_ecfp	This work	Localisation
pZS*24_MCS1	Laboratory collection	(*2*)
pZS-eyfp_tetR	This work	Localisation
pZS-RNaseE_eyfp_tetR	This work	Localisation

Supplementary Table S5. List of DNA primers used in the current work.

Name	Sequence
F_ecfp_ecfp_control_bseSI	ATTAGGGCCCATGGTGAGCAAGGGCGAG
R_ecfp_eyfp_mcherry_bseSI	ATTAGGGCCCTTACTTGTACAGCTCGTCC
F_ecfp_eyfp_C_nudC	ATTAGGGCCCATGGATCGTATAATTGAAAAATTAGATCAC
R_ecfp_eyfp_C_nudC	CAGCTCCTCGCCCTTGCTCTCATACTCTGCCCGACAC
F_eyfp_ecfp	AGCAAGGGCGAGGAGCTG
F_CsdA_ecfp_eyfp_C_bseSI	ATTAGGGCCCATGGCTGAATTCGAAACCACT
R_CsdA_ecfp_eyfp_C	CAGCTCCTCGCCCTTGCTCGCATCGCCACCGAAACG
F_rne_orf_start	ATTAGGGCCCATGAAAAGAATGTTAATCAACGCA
R_rne_C_eyfp	CAGCTCCTCGCCCTTGCTCTCAACAGGTTGCGGACG
L9Fw	ATTAGGGCCCATGCAAGTTATTCTGCTTGAT
L9Rv_ecfpeyfp	CAGCTCCTCGCCCTTGCTTTCAGCTACTACGTTTACGATC
Hfq-XbaI-Fw	CTAGTCTAGAGATGGCTAAGGGGCAATCTTT
Hfq-SmaI-Rv	ATCCCGGGGTTCGGTTTCTTCGCT
pUT18C-Rv	TGGCTTAACTATGCGGCATC
pUC19-dir	GCCAGGGTTTTCCCAGTCACGA
pUC19-rev	GAGCGGATAACAATTTCACACAGG
pACYC184-SacI-Fw	GAGAGCTCGCCGGCGG
pACYC184-Rv2	ACACGGTGCCTGACTGCGTTAG
NudC_prom_Fw	TAATCGATACCAGGCTCTGACAAAAATC
NudC_prom_Rv	GCCAGTTCGGTCATTTTGGCTGTTC
NudC_pZA_Fw	TAGCGGTACCATGGATCGTATAATTGAAAAATTAGATCAC
NudC_pZA_Rv	TATAGGTACCTTACTCATACTCTGCCCGACAC
NudC-Km-Fw	TCCGGCAGACAAGTAATCGA
NudC-Km-Rv	ACGGTGAGGATATCGGAAAA
CsdA-Fw	GTACCATATGGCTGAATTCGAAACCAC
CsdA-Fw4	ATGGCTGAATTCGAAACCAC

CsdA-Rv	AGGATCCTTACGCATCACCACCG
NudC-Fw2	ATGGATCGTATAATTGAAAAATTAG
NudC-Rv-HindIII	TGTTAAGCTTTTGCGGGTAGTAACGCTC
CsdA-HindIII-Fw	CGGAAGCTTGATGGCTGAATTC
CsdA-SacI-Rv	GGAGCTCGGCGCATCA
NudC-Fw	AGAGCCATGGATCGTATAATTGAAAAATTAGATC
NudC-Rv	ATTCCATGGGACAAATCGCCCCCTGC
NudC-Fw-NcoI	TGTAAAGCTTCTCATACTCTGCCCGACAC
CsdA-Fw3	TGGCTGAATTCGAAACCAC
CsdA-444-Pro-Rv	CTTACGCATCTGGCGGTAC
CsdA-Sacl-Rv2	AAGGTACCTCAGTGGTGGTG
CsdA-444-NdeI-Fw	ATACATATGCCGATGCGTCCGAAAC
5S rRNR_N._blot	CGGCGCTACGGCGTTTCACTTCTG
ChiX_N._blot	GCTATTGGCCCGTCAAAGAG
DsrA_N._blot	TTCGTTACACCAGGAAATCTGATGTGTT
GadY_N._blot	AAGAGGATAGTCTGCCGTCTCCAGACTAAT
GcvB_N._blot	CACAACAACACAACATCACAACCGTAAGC
McaS_N._blot	CCAGACTCTACAGTACACACAGCAG
MotR_N._blot	TTGCGCCTCACCGTATCAGTTAAACA
SibD_N._blot	CGTAAGTTTCGCAGCTTATTAACAGCCA
SibE_N._blot	GTTAAGTTTCCCGACTTACTAACAACTC

Supplementary Table S6. List of *E. coli* strains used in the current work.

Strain	Genotype	Source
E. coli BL21(DE3)CodonPlusRIL	F-, *ompT, hsdS(rB–mB–), dcm+, Tetr galλ(DE3), endA, The, [argU ileY leuW] Camr*	*Invitrogen*
E. coli BTH101	F-, *cya-99, araD139, galE15, galK16, rpsL1 (Str r), hsdR2, mcrA1, mcrB1*	*Euromedex*
E. coli BW25113	F-, Δ(*araD-araB*)567, Δl*acZ4787*(::rrnB-3), *λ*-, *rph-1*, Δ(*rhaD-rhaB*)568, *hsdR514*	*Dharmacon*
E. coli BW25113 Δ*nudC*	F-, Δ(*araD-araB*)567, Δ*lacZ4787*(::rrnB-3), *λ*-, *rph-1*, Δ(*rhaD-rhaB*)568, Δ*nudC*767::kan, *hsdR514*	*Dharmacon*
E. coli BW25113 Δ*csdA*	F-, Δ(*araD-araB*)567, Δ*lacZ4787*(::rrnB-3), *λ*-, *rph-1*, Δ(*rhaD-rhaB*)568, Δ*csdA*774::kan, *hsdR514*	*Dharmacon*
E. coli BW25113 *ΔnudC (ΔnudC::*FRT*)*	F-, Δ(*araD-araB*)567, Δ*lacZ4787*(::rrnB-3), *λ*-, *rph-1*, Δ(*rhaD-rhaB*)568, Δ*nudC*767::FRT, *hsdR514*	This work
E. coli BW25113 *ΔnudCΔcsdA*	F-, Δ(*araD-araB*)567, Δ*lacZ4787*(::rrnB-3), λ-, *rph-1*, Δ(*rhaD-rhaB*)568, Δ*nudC*767::FRT, Δ*csdA*774::kan, *hsdR514*	This work

Separate files:

Data S1. Results of UPLC-MSE analysis of NudC interactome.

Data S2. List of NAD-RNAs identified in wt, Δ*nudC* and Δ*csdA* strains using NAD-Seq.

Data S3. Change in gene expression in Δ*nudC* strain compared to wt.

References

1. J. Schindelin, I. Arganda-Carreras, E. Frise, V. Kaynig, M. Longair, T. Pietzsch, S. Preibisch, C. Rueden, S. Saalfeld, B. Schmid, J.-Y. Tinevez, D. J. White, V. Hartenstein, K. Eliceiri, P. Tomancak, A. Cardona, Fiji: an open-source platform for biological-image analysis. *Nat. Methods*. **9**, 676–682 (2012).

2. R. Lutz, H. Bujard, Independent and tight regulation of transcriptional units in Escherichia coli via the LacR/O, the TetR/O and AraC/I1-I2 regulatory elements. *Nucleic Acids Res.* **25**, 1203–1210 (1997).
